# Super-Resolution Microscopy Reveals Specific Recruitment of HIV-1 Envelope Proteins to Viral Assembly Sites Dependent on the Envelope C-Terminal Tail

**DOI:** 10.1371/journal.ppat.1003198

**Published:** 2013-02-28

**Authors:** Walter Muranyi, Sebastian Malkusch, Barbara Müller, Mike Heilemann, Hans-Georg Kräusslich

**Affiliations:** 1 Department of Infectious Diseases, Virology, University Hospital Heidelberg, Heidelberg, Germany; 2 Department of Biotechnology & Biophysics, Theodor-Boveri-Institute, Julius-Maximilians-University, Würzburg, Germany; 3 Institute of Physical & Theoretical Chemistry, Goethe University, Frankfurt am Main, Germany; 4 Bioquant, University of Heidelberg, Heidelberg, Germany; University of Zurich, Switzerland

## Abstract

The inner structural Gag proteins and the envelope (Env) glycoproteins of human immunodeficiency virus (HIV-1) traffic independently to the plasma membrane, where they assemble the nascent virion. HIV-1 carries a relatively low number of glycoproteins in its membrane, and the mechanism of Env recruitment and virus incorporation is incompletely understood. We employed dual-color super-resolution microscopy visualizing Gag assembly sites and HIV-1 Env proteins in virus-producing and in Env expressing cells. Distinctive HIV-1 Gag assembly sites were readily detected and were associated with Env clusters that always extended beyond the actual Gag assembly site and often showed enrichment at the periphery and surrounding the assembly site. Formation of these Env clusters depended on the presence of other HIV-1 proteins and on the long cytoplasmic tail (CT) of Env. CT deletion, a matrix mutation affecting Env incorporation or Env expression in the absence of other HIV-1 proteins led to much smaller Env clusters, which were not enriched at viral assembly sites. These results show that Env is recruited to HIV-1 assembly sites in a CT-dependent manner, while Env(ΔCT) appears to be randomly incorporated. The observed Env accumulation surrounding Gag assemblies, with a lower density on the actual bud, could facilitate viral spread *in vivo*. Keeping Env molecules on the nascent virus low may be important for escape from the humoral immune response, while cell-cell contacts mediated by surrounding Env molecules could promote HIV-1 transmission through the virological synapse.

## Author Summary

Newly formed HIV-1 particles assemble at the plasma membrane of virus producing cells. The inner structural protein Gag and the envelope glycoprotein Env, which are both essential components of infectious virus particles, traffic to the membrane via different pathways. Attached to the inner side of the membrane, Gag assembles into spherical particles that incorporate Env proteins in their surrounding lipid envelope. The mechanism of Env incorporation is incompletely understood, however. Here, we have exploited recently developed super-resolution fluorescence microscopy techniques that yield a near-molecular spatial resolution to analyze HIV-1 Gag and Env distribution patterns at the surface of virus producing cells. We observed recruitment of Env to the surroundings of Gag assembly sites, dependent on the presence of its cytoplasmic domain. A large proportion of Env was found in the vicinity of the Gag assembly sites rather than directly co-localizing with it. These results support an indirect mechanism of Env recruitment, presumably mediated through virus induced changes in the environment of the nascent Gag assembly. Furthermore, they suggest a role for the Env protein in HIV-1 transmission that goes beyond its well-characterized function as an entry protein on the viral surface.

## Introduction

Human immunodeficiency virus type 1 (HIV-1) acquires its lipid envelope by budding through the plasma membrane of an infected cell. Virus morphogenesis is directed by the viral Gag polyprotein, which is sufficient for release of virus-like particles, and also facilitates the incorporation of the viral genome and other important factors including the viral envelope (Env) glycoproteins (reviewed in [1]). Env plays an essential role in virus replication by mediating the fusion between viral and cellular membranes during virus entry. The Env proteins are synthesized as a polyprotein precursor (gp160) that is cleaved to the mature surface glycoprotein gp120 and the transmembrane glycoprotein gp41 by cellular proteases. During virus assembly at the plasma membrane, the gp120/gp41 complex is incorporated into the lipid bilayer of nascent particles as a trimer of heterodimers. Within the virion, these trimeric complexes project from the membrane surface as highly glycosylated spikes (reviewed in [2]). HIV-1 exhibits a relatively low density of glycoprotein spikes on its surface compared to other enveloped viruses with only 7–14 Env complexes per virion [3], [4]. Virus entry is initiated by gp120 binding to the viral receptor CD4 and a chemokine receptor, and completed by insertion of a fusion peptide into the host membrane and conformational changes in gp41 mediating membrane fusion (reviewed in [2]).

HIV-1 Env is transported to the cell surface via the secretory pathway and inserts into the lipid membrane through the gp41 transmembrane domain. However, the mechanism by which the Env glycoprotein complex is incorporated into virus particles remains incompletely understood. Genetic and biochemical evidence points to an important role of the N-terminal, membrane-apposed matrix (MA) domain of the viral Gag polyprotein and the unusually long (151 amino acids) C-terminal tail of gp41 (CT), projecting into the interior of the virion, for Env incorporation [5]–[10]. HIV-1 derivatives lacking their cognate Env proteins can incorporate heterologous viral glycoproteins, thereby adopting their distinctive entry properties, however (‘pseudotyping’; reviewed in [11]). Furthermore, deletion of the Env CT has only minor effects on virion incorporation of Env and viral infectivity in certain permissive cell lines, while strongly reducing Env incorporation and abolishing infectivity in non-permissive cells [12]–[15]. Although these results are consistent with an important role of the CT, they show that an HIV-1 specific signal is dispensible for glycoprotein incorporation.

Four mutually non-exclusive models have been proposed to explain Env incorporation into retroviral particles: (i) random incorporation of plasma membrane proteins including Env, (ii) specific incorporation of Env into the virus by direct protein-protein interactions, (iii) co-targeting of Gag and Env to the same region of the plasma membrane and (iv) indirect incorporation via a bridging factor (reviewed in [2]). There is experimental evidence supporting, or at least consistent with each of these models. Efficient pseudotyping by heterologous glycoproteins and the large number of cellular membrane proteins incorporated into HIV-1 (reviewed in [16], [17]) indicate that there is no exclusion of non-specific proteins. Incorporation may thus be influenced by surface density of the respective protein. A direct interaction between MA and Env CT *in vitro* has been reported [10] and there is strong genetic evidence supporting this interaction [5]–[8], [18]. HIV-1 assembly is believed to occur at specific, raft-like membrane lipid microdomains, and both Gag and Env have been reported to be associated with detergent-resistant membranes (reviewed in [19]). Furthermore, co-expression of HIV-1 Gag with both HIV-1 Env and Ebola virus glycoproteins in the same cells showed efficient incorporation of both glycoproteins, but segregation into separate particle populations suggesting their spatial separation in virus producing cells [20]. Finally, a number of cellular proteins have been found to interact with HIV-1 Env proteins and may act as bridging factors for Env incorporation [2].

Fluorescence microscopy of HIV-1 producing cells shows patchy signals of both Gag and Env at the plasma membrane, while the majority of Env appears to reside in intracellular membrane compartments [21]. Confocal microscopy provided evidence for some colocalisation of Gag and Env at the plasma membrane [21], but reported correlation coefficients are low [20] and the resolution of light microscopy is not sufficient to discern adjacent individual budding sites. Immunostaining of surface glycoproteins and visualization with scanning electron microscopy provided convincing evidence for a specific recruitment of Rous sarcoma virus (RSV) Env proteins to RSV but not to HIV-1 budding sites [22]; this study did not include HIV-1 glycoproteins, however.

Studying the distribution of viral proteins at small spatial scales requires an optical resolution which is beyond the limit of light microscopy (∼200 nm). New super-resolution fluorescence microscopy techniques [23]–[25] have bypassed this resolution limit, providing spatial resolution reaching a near-molecular level. These include single-molecule localization techniques such as photoactivated localization microscopy (PALM) [23] and direct stochastic optical reconstruction microscopy (dSTORM) [26]. PALM and STORM microscopy have been employed to investigate the distribution of viral proteins upon HIV-1 cell entry [27], [28] and to detect Gag assemblies at the plasma membrane [29]–[31]. Lehmann et al. [30] used multicolor super-resolution microscopy to investigate co-localization of the cellular restriction factor tetherin with HIV-1 budding sites. These authors also reported scattered Env distribution at Gag assembly sites, but did not further characterize Env localization [30]. Here, we performed dual-color super-resolution microscopy to analyze HIV-1 Gag and Env distribution patterns in HIV-1 producing cells. We show a CT dependent recruitment of Env to the viral budding site, with Env molecules concentrating around the Gag assemblies and in their periphery.

## Results

For detection of HIV-1 Gag in the viral context, we made use of a construct carrying the photoconvertible protein mEosFP inserted between the MA and capsid (CA) domains of Gag [32]. Proviral constructs carrying a gene encoding an autofluorescent protein at this position yield fluorescently labeled HIV-1 particles with wild-type morphology and infectivity upon co-transfection with equal amounts of their unlabeled counterpart [32]. Since fully assembled HIV-1 buds comprise ∼2,400 molecules of Gag [33], 1,200 molecules of mEosFP are expected to accumulate on average at viral budding sites. In this study we employed constructs based on pCHIV [34] that encodes all HIV-1_NL4-3_ proteins except Nef, but is replication deficient due to deletion of the viral long terminal repeats. The ratio of Env to Gag in transfected cells is thus comparable to that found in HIV-1 infected cells. Env molecules at the cell surface were detected by indirect immunolabeling using a human monoclonal antibody against gp120 (MAb 2G12; [35]). Primary antibody binding was revealed by a secondary antibody coupled to Alexa Fluor 647 followed by dSTORM imaging. Due to the higher photon yield of organic fluorophores, dSTORM imaging yields improved single-molecule localization and thus higher spatial resolution than PALM. The combination of PALM for detection of the abundant Gag molecules with a known lattice structure [1] and dSTORM for detection of the much rarer Env molecules [3] thus appeared ideally suited for the purpose of this study.

**Figure 1:**
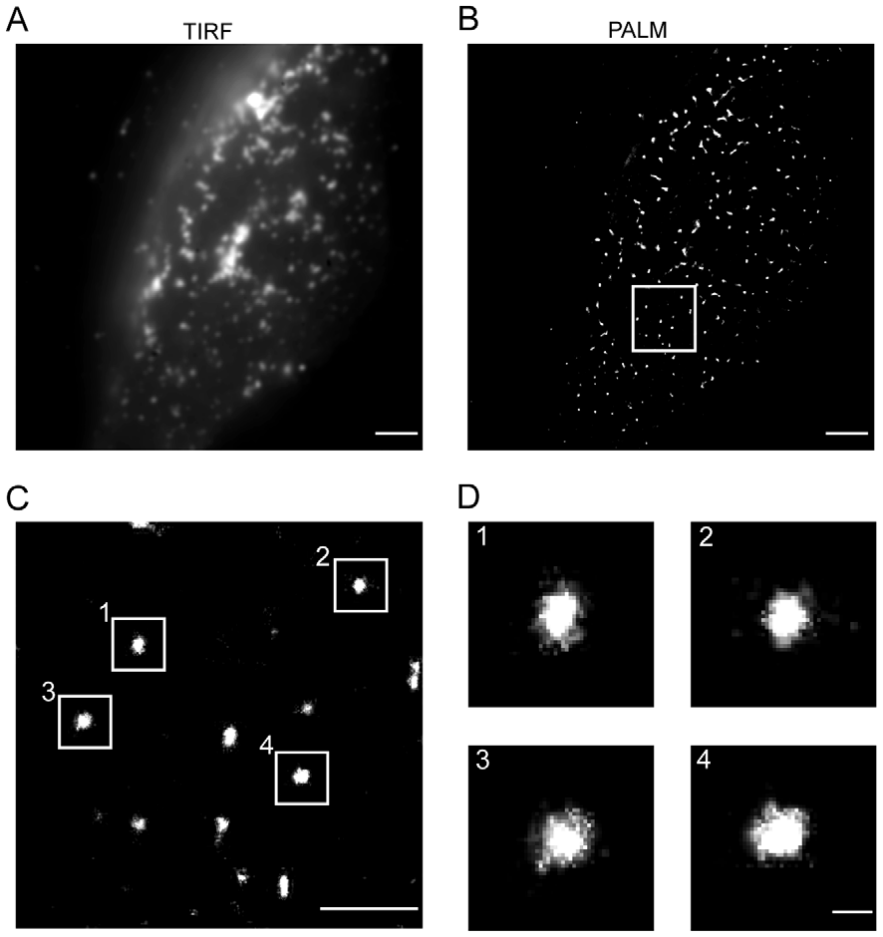
Distribution of Gag.mEosFP at the plasma membrane of HeLa cells imaged by super-resolution TIRF microscopy. HeLa cells were transfected with equimolar amounts of pCHIV and pCHIV^mEosFP^. At 24 hpt, cells were fixed and HIV-1 assembly sites were imaged by PALM as described in Materials and Methods. Summed intensity TIRF (**A**) and high resolution PALM (**B**) microscopy images of a representative cell are displayed. Scale bars correspond to 2 µm. The boxed region in (B) corresponds to the region of interest shown as an enlarged high resolution image in (**C**). Scale bar in (C) corresponds to 1 µm. (**D**) Four individual HIV-1 assembly sites from the boxed regions shown in (C). Scale bar corresponds to 100 nm.

Total internal fluorescence (TIRF) microscopy and TIRF-PALM imaging of Gag.mEosFP at the plasma membrane of transfected HeLa cells was performed for detection of HIV-1 assembly sites. At the conditions used, Gag.mEosFP was detected with a localization accuracy of ∼28 nm in PALM mode (Figure S1). TIRF microscopy revealed punctuate structures of Gag.mEosFP at the plasma membrane as described previously for Gag.eGFP [36] (Figure 1A). These punctae could be clearly resolved into individual assembly sites by super-resolution imaging (PALM; Figure 1B–D). Compact round assemblies with a diameter of ∼130 nm (Figure 1D), closely resembling assembly sites detected by dSTORM or PALM in previous studies [29], [30] were considered to represent HIV-1 budding structures. The PALM resolution achieved here was not sufficient to detect the semi-spherical architecture of individual Gag shells, which we have recently shown at higher resolution (∼18 nm) by dSTORM imaging of Gag.SNAP assembly sites labelled with a bright synthetic fluorophore [31].

**Figure 2:**
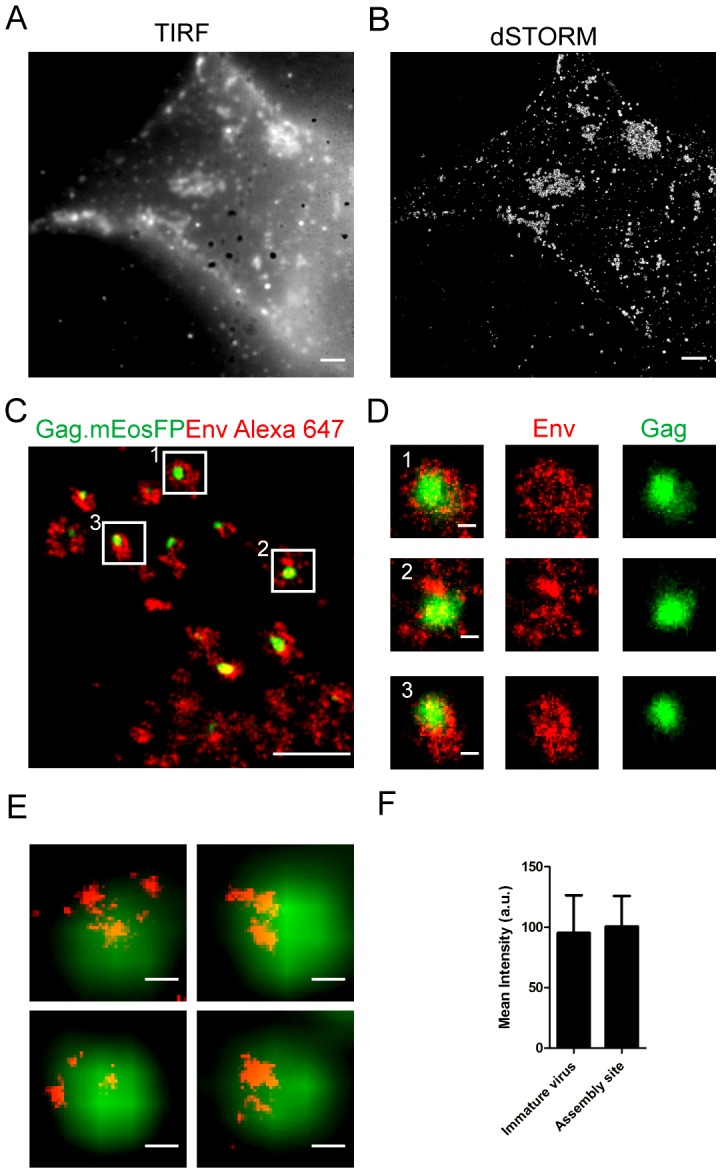
Distribution of HIV-1 Env at the plasma membrane of HeLa cells visualized by super-resolution TIRF microscopy. HeLa cells were transfected with equimolar amounts of pCHIV and pCHIV^mEosFP^. Cells were fixed 24 hpt, stained by indirect immunofluorescence using MAb 2G12 and goat anti-human Alexa Fluor 647 and imaged by dSTORM as described in Materials and Methods. Summed intensity TIRF (**A**) and high resolution dSTORM (**B**) microscopy images of a representative cell are displayed. Scale bars correspond to 2 µm. (**C**) Region from the plasma membrane of a representative cell, showing the superposition of a PALM image for Gag.mEosFP (green) and the corresponding dSTORM image of Env stained with Alexa Fluor 647 (red), respectively. HeLa cells transfected with equimolar amounts of pCHIV and pCHIV^mEosFP^ were fixed and stained by indirect immunofluorescence using MAb 2G12 and goat anti-human Alexa Fluor 647. Cells were subjected to dual-color super-resolution microscopy as described in Materials and Methods. Scale bar corresponds to 1 µm. (**D**) Individual HIV-1 assembly sites from the boxed regions are shown. Scale bar corresponds to 100 nm. (**E**) Env surface distribution on extracellular immature HIV-1 particles. Particles were purified from the supernatant of 293T cells transfected with pCHIV carrying a point mutation in the HIV-1 protease active site [39]. An eGFP-tagged version of the accessory protein Vpr [63] was included as a marker to localize the position of individual virions in diffraction limited images. Virions were adhered to fibronectin-coated coverslips, fixed and immunostained with MAb 2G12 and goat anti-human Alexa Fluor 647. The panel shows images of four individual particles with the eGFP signal (green) recorded in diffraction limited mode and the Alexa Fluor 647 (red) signal recorded by dSTORM. Scale bars correspond to 100 nm. (**F**) Comparison of the Env signal intensities on single extracellular immature particles with the Env signal intensities colocalizing with HIV-1 assembly sites defined by the dSTORM signal for Gag.mEosFP. Fluorescence intensities of the Alexa Fluor 647 signals were determined for the super-resolution images of ten individual particles each. The histogram shows the average values and SD of the fluorescence intensity.

HeLa cells transfected as above were fixed and subjected to immunostaining for HIV-1 Env, followed by TIRF- and TIRF-dSTORM microscopy. Alexa Fluor 647, coupled to the protein of interest through the primary and secondary antibody, was detected with a localization accuracy of ∼15 nm in dSTORM mode (Figure S2). TIRF microscopy showed a multi-clustered Env distribution (Figure 2A), similar to previously reported results [21]. Env clusters of various sizes were observed by dSTORM imaging (Figure 2B). These clusters appeared larger and less compact than the Gag.mEosFP assemblies detected in the same cells (compare Figure 1). Dual-color super-resolution microscopy was performed on HeLa cells expressing HIV^mEosFP^ to determine the relative localization of Env with respect to viral Gag assemblies. As shown in the representative images in Figures 2C and 2D, Env clusters surrounding Gag assembly sites were often round and sometimes displayed a doughnut-like shape. A similar pattern was observed when another antibody against gp120 (MAb b12, [37]) was used (Figure S3), or when a harsher fixation protocol reported to block membrane protein motility [38] was applied (Figure S4). Env clusters of similar morphology were also observed in regions lacking a detectable Gag.mEosFP signal (Figure 2C), and only ∼50% of Env clusters were associated with obvious HIV-1 budding sites. Env clusters not associated with characteristic Gag assemblies may correspond to early budding sites with a low number of Gag molecules. Furthermore, co-transfection with a wt plasmid encoding unlabeled Gag was performed in our experiments, and some assembly sites may thus contain a low number of Gag.mEosFP molecules. To address this issue, HeLa cells were transfected with pCHIV^mEosFP^ alone and subsequently processed and analyzed as above. Similar Gag assembly sites surrounded by larger Env clusters were observed, but ∼90% of Env clusters were found to be associated with Gag assemblies in this case (Figure S3).

Parallel control experiments showed that omitting the primary antibody from the staining solution or analysis of cells expressing a virus derivative lacking the viral Env protein yielded only few dense Alexa Fluor 647 signals, while the Env clusters were completely lost (Figure S5). The remaining non-specific signals presumably represent aggregates of secondary antibody. Env clusters overlapping HIV-1 budding sites showed a scattered distribution and always extended beyond the area, in which Gag.mEosFP molecules were detected (Figure 2C, D). Furthermore, the density of Env localization events was not enriched at the position of the Gag.mEosFP assembly compared to the directly adjacent membrane area, but often appeared lower over the actual Gag assembly site (Figure 2C, D; see below). A comparative quantitative analysis of the Env signal on budding sites and released immature virions was performed to determine whether the comparatively low proportion of Env signals directly co-localizing with Gag constitutes the full complement of Env on the virion or whether peripheral Env clusters also contribute to the Env signal on the virus. Immature virions were used for this comparison, since the Gag lattice at the budding site is also immature and the Env distribution pattern on the viral surface is altered by proteolytic maturation of the inner virion structure [39]. Purified immature viral particles were fixed, stained with 2G12 as described for virus producing HeLa cells, and analyzed by dSTORM. Env signals distributed in multiple clusters were detected on the viral surface (Figure 2E) in agreement with recent results obtained by stimulated emission depletion microscopy [39]. Quantitative assessment revealed a similar intensity of the total Env signal on extracellular immature virions compared to the Env signal directly co-localizing with the Gag assembly site (Figure 2F). Accordingly, the Env content of the virion appears to be derived from the relatively low number of Env molecules directly overlapping the Gag assembly sites, while the larger Env cluster surrounding nascent assembly sites is not incorporated into the virion. Env recruitment was also investigated in the A3.01 T-cell line, since T-cells represent a natural target cell of HIV-1. A similar distribution of Gag assembly sites with overlapping Env clusters, but increased Env density at the periphery and surrounding the actual budding site was observed by super-resolution dual-color imaging of A3.01 cells producing HIV-1^mEosFP^ (Figure S6A, 6B).

**Figure 3:**
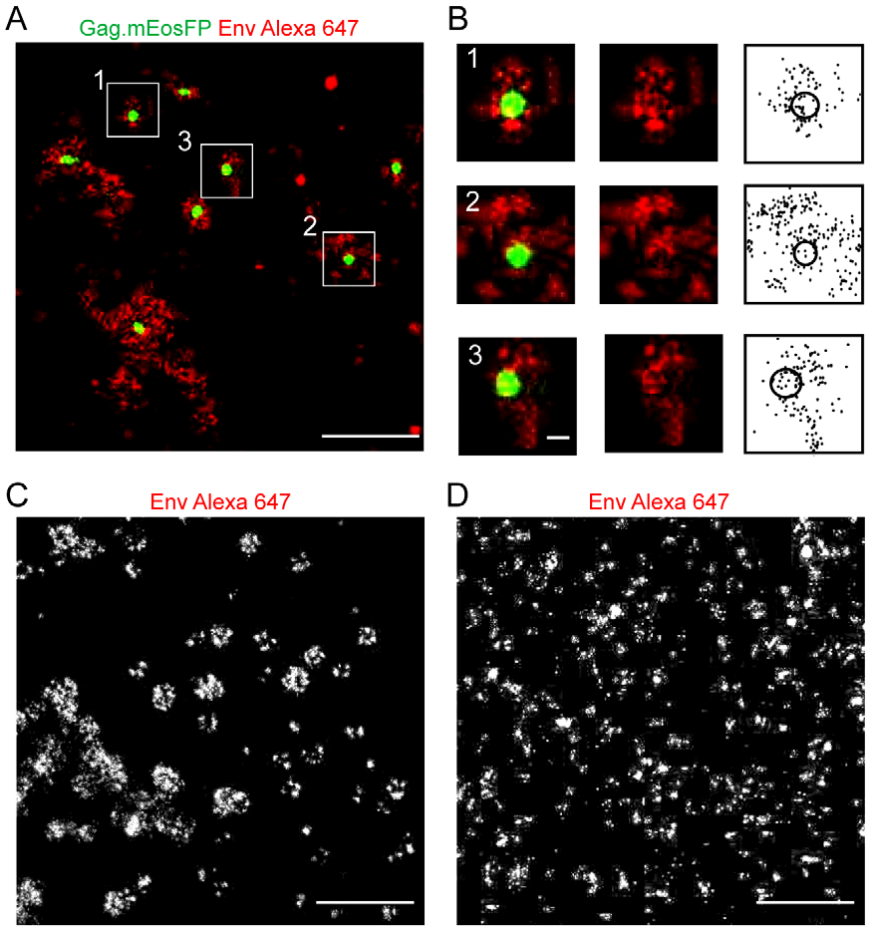
Comparative analysis of Env(wt) distribution expressed in the viral context or alone. (**A, B**) Distribution of HIV-1 Gag and Env at the plasma membrane of HeLa cells transfected with equimolar amounts of pCHIV and pCHIV^mEosFP^. Unfixed cells were stained by indirect immunofluorescence using Fab 2G12 and Fab goat anti-human Alexa Fluor 647, fixed, and subjected to dual-color super-resolution microscopy as described in Materials and Methods. (**A**) Region from the plasma membrane of a representative cell, showing the superposition of a PALM image for Gag.mEosFP (green) and the corresponding dSTORM image of Env stained with Alexa Fluor 647 (red), respectively. Scale bar corresponds to 1 µm. (**B**) Enlargement of three individual assembly sites from the boxed regions indicated in (A). The figure shows merged super-resolution images (left panels), the dSTORM Env Alexa Fluor 647 image (middle panels) and individual Alexa Fluor 647 localizations from all images recorded in the defined area as black dots, with a black circle representing the rims of the Gag cluster (right panels), respectively. Scale bar corresponds to 100 nm. (**C, D**) Env distribution patterns in the presence (C) and absence (D) of other HIV-1 derived proteins. HeLa cells were transfected with equimolar amounts of pCHIV and pCHIV^mEosFP^ (**C**) or with pEnv(wt) (**D**), respectively. Unfixed cells were stained with 2G12 Fab and Alexa Fluor 647 Fab, fixed, and visualized by dual-color super-resolution microscopy as described in Materials and Methods. Scale bars represent 1 µm.

The experiments described were carried out employing fixed cells stained with complete IgG molecules, which could potentially affect distribution and detection of the molecules of interest. To exclude potential artifacts, all further experiments (except for those with the MA(mut) constructs, see below) were performed using unfixed cells (stained at 16°C to prevent membrane protein internalization) and immunostaining with Fab fragments. The distribution pattern for Gag assembly sites and Env molecules was largely unaltered when HeLa cells expressing HIV^mEosFP^ were analyzed in this way (Figure 3A) compared to the initial protocol (Figure 2C). Tightly packed clusters of Gag.mEosFP with a diameter of ∼130 nm marked HIV-1 assembly sites, which were overlayed by less dense Env clusters that commonly exhibited enrichment in the periphery and surrounding the Gag budding site (Figure 3B). To investigate whether the membrane distribution of HIV-1 Env was determined by the Env protein itself or was altered by the co-expression of other viral proteins, comparative dSTORM microscopy was performed on HeLa cells expressing either HIV^mEosFP^ as before or only HIV-1 Env in the absence of other viral proteins. Super-resolution imaging of Env alone yielded a clearly different pattern compared to Env in the viral context. The large and distinctive round or doughnut shaped Env clusters, which frequently marked HIV-1 assembly sites (Figure 3C), were not seen in the absence of other viral proteins. Instead, much smaller and more dispersed clusters were observed when Env was expressed alone (Figure 3D). These results demonstrate a clear influence of the other HIV-1 proteins, most likely Gag, on the membrane distribution of HIV-1 Env.

**Figure 4:**
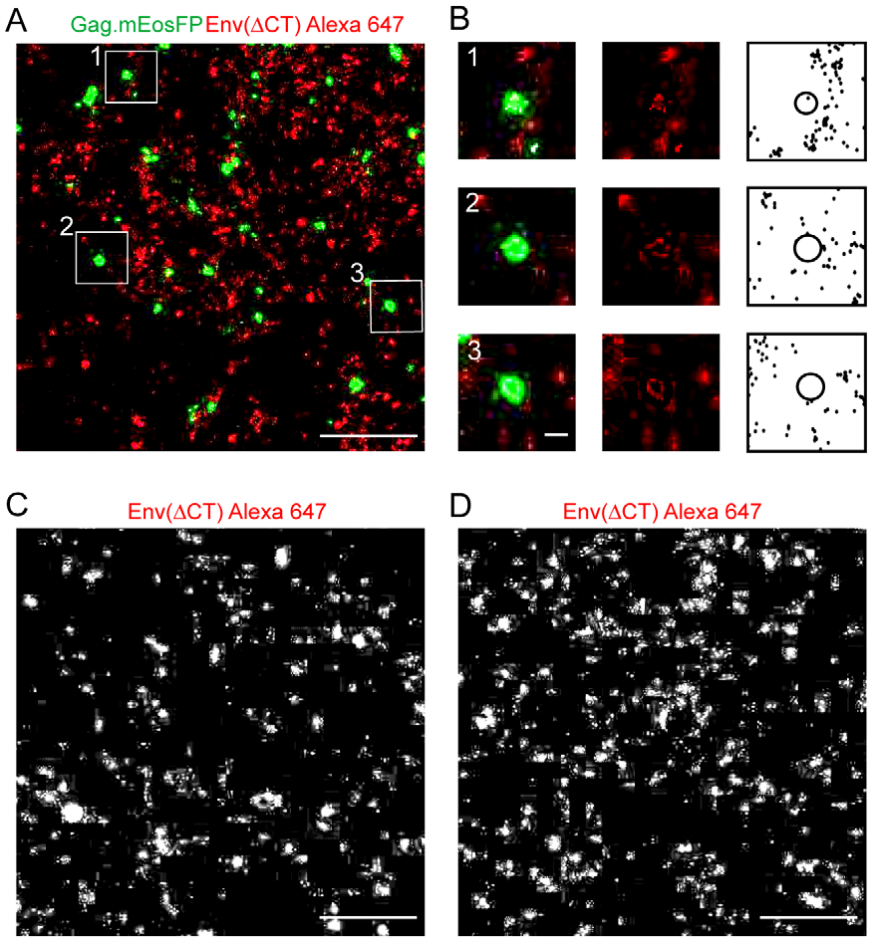
Comparative analysis of Env(ΔCT) distribution expressed in the viral context or alone. (**A, B**) Distribution of HIV-1 Gag and Env at the plasma membrane of HeLa cells transfected with equimolar amounts of pCHIV.Env(ΔCT) and pCHIV^mEosFP^. Env(ΔCT). Unfixed cells were stained by indirect immunofluorescence using Fab 2G12 and Fab goat anti-human Alexa Fluor 647, fixed, and subjected to dual-color super-resolution microscopy as described in Materials and Methods. (**A**) Region from the plasma membrane of a representative cell, showing the superposition of a PALM image for Gag.mEosFP (green) and the corresponding dSTORM image of Env(ΔCT) stained with Alexa Fluor 647 (red), respectively. Scale bar corresponds to 1 µm. (**B**) Enlargement of three individual assembly sites from the boxed regions indicated in (A). The figure shows merged super-resolution images (left panels), the dSTORM Env Alexa Fluor 647 image (middle panels) and individual Alexa Fluor 647 localizations from all images recorded in the defined area as black dots, with a black circle representing the rims of the Gag cluster (right panels), respectively. Scale bar corresponds to 100 nm. (**C, D**) Comparison of Env(ΔCT) distribution patterns in the presence (C) and absence (D) of other HIV-1 derived proteins. HeLa cells were transfected with equimolar amounts of pCHIV.Env(ΔCT) and pCHIV^mEosFP^Env(ΔCT) (**C**) or with pEnv(ΔCT) (**D**), respectively. Unfixed cells were stained with 2G12 Fab and Alexa Fluor 647 Fab, fixed, and visualized by dSTORM as described. Scale bars represent 1 µm.

To investigate whether the long Env CT and its presumed interaction with the underlying Gag lattice are important for Env distribution on the surface of HIV-1 producing cells, we performed dual-color super-resolution microscopy on HeLa cells expressing HIV^mEosFP^Env(ΔCT). Tightly packed clusters of Gag.mEosFP as described above were detected in this case as well and identified *bona fide* HIV-1 budding sites (Figure 4A). Clusters of Env(ΔCT) appeared much smaller than observed for wild-type (wt) Env, however. In contrast to (wt) Env clusters, Env(ΔCT) clusters were not enriched at HIV-1 budding sites, but appeared to be more randomly distributed (Figure 4A, B). Furthermore, the characteristic Env clusters observed for the wt protein (compare Figure 3C) were not observed in this case. Similar results were obtained in HIV^mEosFP^ expressing A3.01 cells (Figure S6). Comparison of Env distribution patterns in cells expressing either HIV^mEosFP^Env(ΔCT) (Figure 4C) or only Env(ΔCT) (Figure 4D) using super-resolution microscopy revealed no apparent difference with similar cluster size and distribution in both cases. Thus, the characteristic Env distribution appeared to depend on the presence of other viral proteins and on the Env CT.

**Figure 5:**
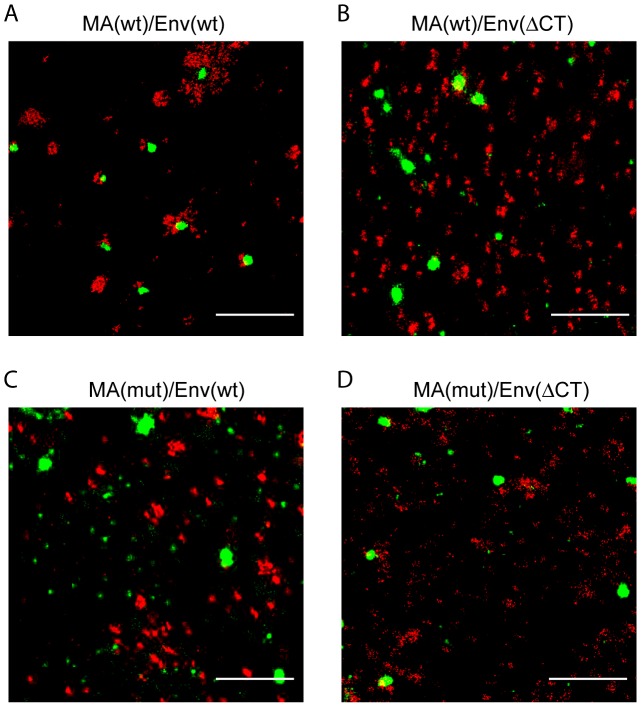
Distribution of Env(wt) or Env(ΔCT) in the context of an Env-interaction deficient Gag variant. HeLa cells were transfected with proviral constructs carrying both wt Gag and wt Env (**A**), wt Gag and Env(ΔCT) (**B**), Gag carrying the MA mutation and wt Env (**C**), or comprising both mutated MA and Env(ΔCT) (**D**), respectively. Fixed cells were stained by indirect immunofluorescence using MAb APR342 and goat anti-mouse Alexa Fluor 532 and MAb 2G12 and goat anti-human Alexa Fluor 647, and subjected to dual-color dSTORM. Scale bar corresponds to 1 µm. An overview of respective cells is presented in Figure S7.

The most likely viral protein responsible for the observed specific Env distribution pattern in the full viral context is Gag since several mutations in its MA domain have been shown to affect Env incorporation [5]–[7], [9]. To directly address this issue, we made use of a panel of proviral constructs carrying either the wt sequence or two point mutations (L8S/S9R) within the MA domain of Gag [8], [40] (designated here as MA(mut)) in the context of Env(wt) or Env(ΔCT). These point mutations have been shown to affect Env(wt) particle incorporation and thereby viral infectivity; both defects are alleviated by truncation of the Env CT [8]. Transfected HeLa cells were fixed and immunostained with antibodies against MA and Env followed by dual-color dSTORM analysis (Figure 5, S7). Typical Gag assembly sites were readily detected in all cases and were associated with Env clusters extending beyond the assembly site for the wt construct (Figure 5A, S7A). These Gag associated Env clusters were absent for MA(wt) in the context of Env(ΔCT) (Figure 5B, S7B) consistent with the results observed above. A similar phenotype was observed for Env(wt) in the context of the MA(mut) virus (Figure 5C, S7C), indicating that the reported Env incorporation defect is due to a loss of Env recruitment to the assembly site. The combination of both mutations displayed an intermediate phenotype, with the overall Env distribution pattern (Figure S7D) resembling the pattern observed for the individual MA or Env mutants, but a slightly more pronounced Env-Gag co-localization revealed upon inspection of individual assembly sites (Figure 5D).

**Figure 6:**
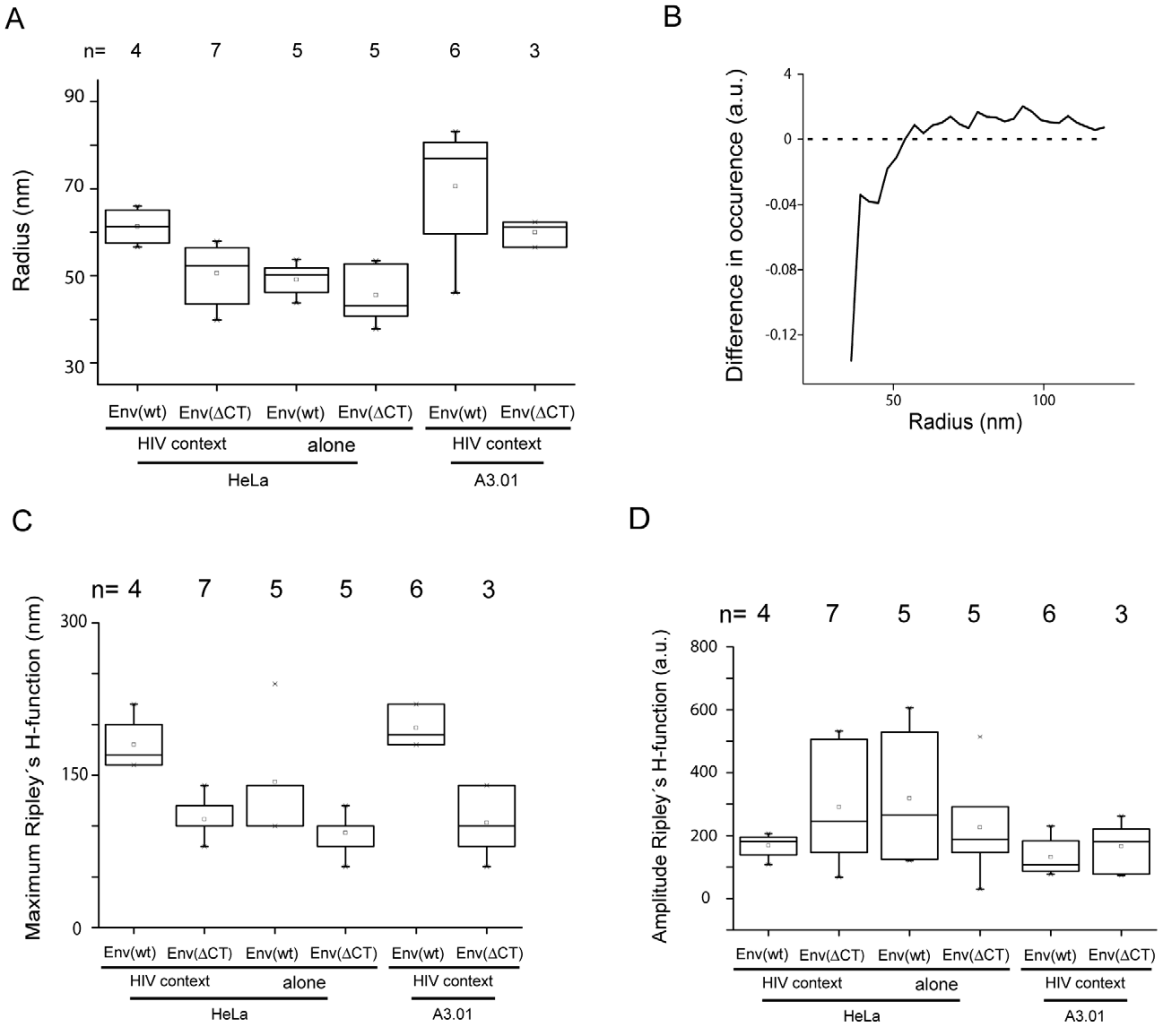
Computational cluster analysis of HIV-1 Env membrane distribution. Global cluster analysis of Env distribution at the plasma membrane was performed based on super-resolution images of HeLa cells transfected with pCHIV/pCHIV^mEos.FP^, pCHIV. Env(ΔCT)/pCHIV^mEosFP^Env(ΔCT), pEnv(wt) or pEnv(ΔCT), or of A3.01 T-cells nucleofected with pCHIV/pCHIV^mEos.FP^ or pCHIV.Env(ΔCT)/pCHIV^mEosFP^Env(ΔCT), respectively. (**A**) Image-based, morphological cluster analysis of entire cells. (**B**) Differential distribution of cluster size for Env(wt) on the surface of HeLa cells in the presence and absence of other viral proteins. The curve was derived by subtraction of the normalized distribution of cluster size obtained by image-based cluster analysis for Env(wt) expressed in the HIV-1 context from that obtained upon expression of Env(wt) alone. (**C**) Coordinate based all-distance distribution analysis by Ripley's H-function. Statistical evaluation of the maximal H-values [nm] was performed as a correlate of the average cluster diameter. (**D**) Statistical evaluation of the amplitude of the H-function [a.u.] as a measure of the average degree of clustering. Box plots display 5^th^ percentile, 25^th^ percentile, median (straight line), mean (square), 75^th^ percentile and 95^th^ percentile.

To describe the different Env distribution patterns at the cell membrane in an objective and quantitative manner, we performed mathematical cluster analysis using two complementary approaches. Data sets were derived from transfected HeLa or A3.01 cells, respectively; at least three cells per condition were included in the analysis. In a first approach, we used an image-based morphological cluster analysis (Figure 6A) to determine the average cluster size of Env in whole cells. The average cluster size of Env(wt) in virus producing cells was significantly larger than observed for Env(wt) expressed alone or for clusters formed in cells expressing Env(ΔCT) (with or without other viral proteins). The distribution of cluster sizes of Env(wt) in HeLa cells was also analyzed by subtracting values obtained for cells expressing only Env from those obtained for HIV-1 producing cells (Figure 6B). Positive values in this analysis indicate an enrichment of the respective cluster size in virus-producing cells. This analysis revealed that smaller clusters with a radius <50 nm are much more prominent in cells expressing only Env, while larger clusters with a radius of 50 to 150 nm predominate in virus-producing cells.

In an independent second approach, a coordinate-based distance distribution analysis using Ripley's H-function [41] was applied (Figure 6C, 6D and S8). This approach compares the measured distribution of single-molecule localizations to a simulated random distribution, and provides information whether clustering occurs. The maximum of the H function reflects the average size of clusters. The amplitude of the H-function is a measure of the degree of clustering. The variance of the amplitude reflects the variance of the spatial organization of proteins within a cluster. Unbiased analysis of clustering confirmed the larger size of Env(wt) clusters in virus producing cells compared to expression of Env alone or Env(ΔCT) (with or without other viral proteins) (Figure 6C). Furthermore, clusters of Env(wt) in HIV-1 producing cells exhibited a much smaller variation in cluster size (Figure S8) and a higher degree of homogeneity of clustering (Figure 6D) than observed for any of the other conditions. This result shows that Env(wt) clusters in HIV-1 producing cells are mostly homogenous and organized and most Env(wt) molecules are likely to be found in a single type of arrangement. The results of both cluster analyses were similar for HeLa and A3.01 cells, and corroborated the results of visual inspection of the spatial distribution of HIV-1 Env. Because whole cells were analyzed in this case and no pre-selection of particular regions was made, the results of the computational analyses represent average values and provide a more general and unbiased pattern.

**Figure 7:**
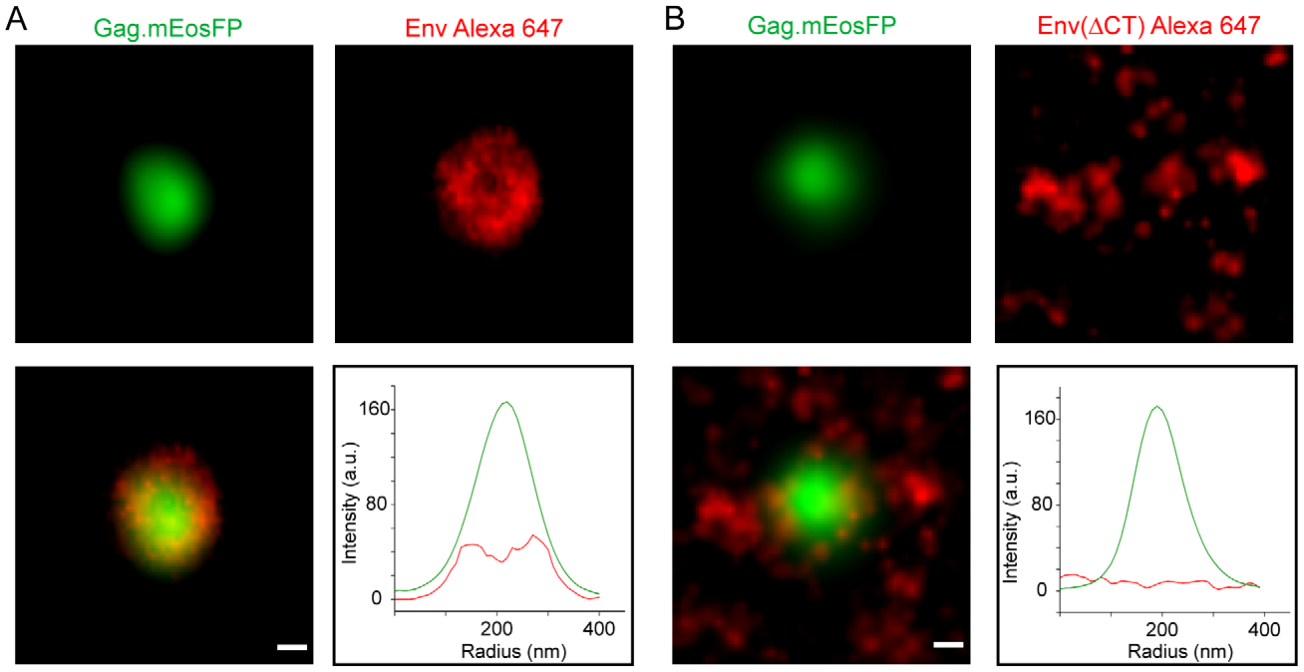
Averaged super-resolution intensity distribution of Env at HIV-1 assembly sites. Image-based, averaged assembly site analysis performed on super-imposed high-resolution images of individual Gag.mEosFP clusters (green) from cells transfected with pCHIV/pCHIV^mEosFP^ (**A**) or pCHIV.Env(ΔCT)/pCHIV^mEosFP^Env(ΔCT) (**B**), respectively (n = 7 assembly sites per condition) and the averaged Env Alexa Fluor 647 signal in the respective area (red). Graphs show the profile of intensity through the center of the averaged, overlaid intensity images: green line, Gag.mEosFP; red line, Env. Scale bars represent 50 nm.

While providing a view on the whole cell, these computational analyses were not suitable to decipher differences in the Env distribution pattern at specific localizations, e.g. HIV-1 budding sites. To obtain quantitative information on the spatial distribution of Env at such sites, it was required to analyze preselected regions. This was performed by aligning and averaging the intensity distribution of Gag.mEosFP and Env at seven HIV-1 assembly sites each from HeLa cells producing either HIV^mEosFP^ or HIV^mEosFP^Env(ΔCT). This procedure should enhance features common to the respective assembly sites, while deemphasizing random distributions. The averaged images of assembly sites from cells producing HIV-1 containing Env(wt) or Env(ΔCT), respectively, showed a very similar pattern for the respective Gag assembly sites but a dramatic difference for the Env distribution pattern (Figure 7). The full-length Env protein (Figure 7A) exhibited a doughnut-shaped average pattern, reflected by a bimodal distribution of Env with a local intensity minimum at the position of the peak of the Gag intensity distribution (Figure 7A). In contrast, Env(ΔCT) (Figure 7B) displayed a more random distribution without any significant enrichment at or close to the actual budding site. The averaged intensity profiles recorded for cells producing the Env(ΔCT) virus revealed no distinctive pattern of Env and no enrichment of Env density in the region of the Gag assembly site (Figure 7B), again confirming the qualitative results obtained by visual inspection of the images.

## Discussion

Here, we employed dual-color super-resolution microscopy to investigate the distribution of HIV-1 Env glycoproteins at the cell surface of virus-producing and Env expressing cells. A combination of PALM imaging of an autofluorescent HIV-1 Gag derivative with dSTORM imaging of Env immunostained with a fluorescent dye appeared to be the optimal strategy. In general, synthetic fluorophores have a higher photon yield than fluorescent proteins, thereby providing increased spatial resolution in single-molecule based super-resolution imaging [26]. Accordingly, dSTORM imaging of Gag assemblies had revealed spherical edge effects [31] which were obscured in previous PALM images of Gag fused to autofluorescent proteins [30]. However, the structure of the Gag lattice at HIV-1 budding sites has already been characterized by electron tomography [33], and Gag staining with synthetic fluorophores requires cell fixation and immobilization that might affect Env protein distribution. In contrast, the cell surface protein Env can be detected on native cells by immunolabeling and subsequent detection using organic fluorophores. Thus, we applied PALM for detection of Gag, while the higher localization accuracy of dSTORM was exploited for the detailed characterization of Env surface distribution.

Using this system, we have analyzed Env localization in the presence and absence of other viral proteins as well as for a variant with mutations in the MA region of Gag and determined the role of the CT for HIV-1 Env membrane distribution. The results clearly revealed a CT- and MA dependent recruitment of Env proteins to the vicinity of Gag assembly sites. Env proteins accumulated around Gag clusters, concentrating at their periphery, while the bud center displayed reduced Env density. These results are consistent with Gag-dependent Env accumulation surrounding individual sites of HIV-1 particle formation, while the observed larger extension of Env clusters compared to that of Gag assemblies suggests that other factors than direct Gag-Env interaction may contribute to Env recruitment.

Comparing Env distribution on cells producing HIV-1 particles with the distribution of Env expressed in the absence of other viral components revealed a difference that was clearly recognizable even without co-detection of the HIV-1 Gag protein. A scattered membrane distribution of Env with small clusters was seen in cells expressing Env alone, while larger accumulations were detected in virus producing cells. The appearance of the larger clusters was dependent on the Env CT and was disrupted by a MA mutation that abolishes Env incorporation. Dual color analysis revealed that larger Env clusters were commonly (∼90%) associated with *bona fide* HIV-1 assembly sites, and Env structures with the characteristic round or doughnut-like shape were absent in cells expressing Env alone. The remaining ∼10% of larger Env clusters apparently lacking Gag association could correspond to nascent HIV-1 assembly sites with a low number of Gag molecules or constitute remnants of prior assembly sites after extracellular release of the viral particle. Alternatively, they could be induced by HIV-1 mediated changes of the membrane environment leading to membrane areas conducive for Env accumulation.

Unbiased computational intensity-based cluster analysis from whole-cell data confirmed that the average cluster size was largest for Env(wt) in the presence of other HIV-1 proteins. This was supported by subtractive distribution of cluster sizes showing that Env(wt) expressed alone was mainly found in small clusters (r<50 nm), whereas the same protein formed clusters with a radius of 50 to 150 nm in HIV-1 producing cells. While image-based cluster analysis can be performed on whole-cell data and generates distributions of cluster sizes, it depends on the spatial resolution and pixel size of the image and requires setting intensity thresholds. Thus, we applied Ripley's H-function [41] as a complementary approach for cluster analysis. This method can only analyze regions of interest rather than whole cells and does not provide information on the distribution of cluster sizes, but it is independent of thresholds. The maximum of the H-function, which correlates with the average cluster size, again demonstrated that the largest clusters were formed by Env(wt) in HIV-1 producing cells. In addition, Ripley's-H function provides information on the heterogeneity of clustering through the variance of its amplitude. A very small variance was observed for Env(wt) in HIV-1 producing cells suggesting a single type of cluster. The sharper peak of the H-function in this case compared to the other three conditions (Figure S8) further indicated more regular clustering. Thus, evaluation of the data sets by two independent types of computational cluster analysis yielded a complete and consistent picture, confirming and extending the results of visual inspection of the super-resolution images.

Considering the four described models for Env incorporation, the reported data clearly argue for specific recruitment and against random incorporation for wt HIV-1. In contrast, random incorporation appears to be likely for Env(ΔCT). Averaging the Env signal at multiple HIV-1 budding sites revealed no distinctive features for the Env(ΔCT) virus, confirming the visual impression. Env(ΔCT) incorporation into HIV-1 particles may thus occur randomly and only depend on its cell surface concentration. This interpretation is consistent with its observed reduced incorporation compared to Env(wt) [7], [9] and the cell type dependence of the mutant phenotype [12]–[15]. Loss of apparent accumulation of Env at viral budding sites was also observed when a RSV variant with a truncated Env CT was analyzed by scanning electron microscopy of immunostained cells [22]. Thus, CT dependent specific recruitment of Env to the assembly site may be a general feature of retroviruses rather than being determined by the long lentiviral CT. Random incorporation may also be expected for pseudotyping with other viral glycoproteins. However, Jorgenson et al. [22] reported accumulation of at least some heterologous viral glycoproteins at RSV and HIV assembly sites, arguing for specific recruitment even in the absence of the cognate Gag-Env pair.

Genetic data from multiple studies support a direct interaction of the HIV-1 Env CT with the membrane-apposed MA layer [6]–[9], [18] even though biochemical evidence for this interaction is weak. Here, we observed that a mutation in MA that had been shown to disrupt virion incorporation of Env [8], [40] abolished formation of Env clusters at HIV-1 assembly sites. This observation is consistent with MA-dependent recruitment of Env to the viral budding site. On the other hand, Env clusters in the case of wt HIV-1 significantly extended beyond the respective Gag assembly sites. Even close inspection revealed no detectable enrichment of Gag signals surrounding *bona fide* budding sites, consistent with the proposition that recruitment of Gag to the nascent bud occurs mostly from a cytoplasmic pool [36]. The majority of Env signals within the respective cluster were detected in the region surrounding the bud, however. Thus, Gag density in the vicinity of HIV-1 budding sites did not appear to differ significantly from its density in other plasma membrane regions, while Env density clearly did. Furthermore, Env density within the cluster appeared lowest at the center of the viral bud, where Gag is most concentrated. These results do not rule out a direct Gag-Env interaction, but argue that an indirect mode may at least contribute to Env concentration at HIV-1 assembly sites. This function clearly depends on HIV-1 Gag since Env accumulation was not seen in the absence of other viral proteins or in the case of the MA mutant. Co-targeting of Gag and Env to pre-existing membrane microdomains with special properties could therefore also not explain our results. Conceivably, accumulation of HIV-1 Gag and possibly of other viral proteins may induce an altered membrane micro-environment which attracts Env(wt) molecules. Alternatively, Env may be recruited by a proteinaceous bridging factor, which directs the viral glycoproteins to the assembly site, but does not immobilize them at this position. A specific membrane environment at the viral budding site is consistent with the raft-like lipid composition of HIV-1 particles [42], [43] and the association of budding sites with tetraspanin-enriched microdomains (reviewed in [44]). The hypothesis that Gag assembly at the plasma membrane alters or induces a specific lipid environment is also consistent with results from several recent studies using fluorescence recovery after photobleaching, fluorescence resonance energy transfer or antibody co-patching of membrane proteins to reveal Gag dependent changes in marker protein mobility or distribution (reviewed in [45]). Dual- or triple-color super-resolution microscopy using marker membrane proteins or lipid dyes could in the future provide direct evidence regarding the size and colocalization of such domains with viral Gag and Env clusters, and may further clarify the mechanism of Env recruitment.

At first glance, the accumulation of large amounts of Env surrounding, but not co-localizing with, Gag assemblies appears surprising for viral production sites. The observed bimodal shape of the Env density profile with a minimum in the central region of the HIV-1 bud is consistent with the low density of Env trimers detected on HIV-1 virions [3], [4], however, and may be explained by limited compatibility of the trimeric CT with the tightly packed Gag lattice. Many cellular membrane proteins are incorporated into HIV-1 particles [16], [17], but efficiency dependent on the CT length has been described, suggesting steric hindrance [46], and exclusion due to interaction of CTs with cytoplasmic factors has been observed [47]. The low number of viral glycoproteins on the particle surface has been suggested to provide a selective advantage by allowing escape of the virus from the humoral immune response [48]. Concentrating surplus Env proteins not required for infectious particle formation around viral buds, on the other hand, could play an important role in forming and maintaining the virological synapse during cell-to-cell transmission. The formation of contacts between infected and uninfected T-cells is induced by Env [49], and viral spread through such synapses is considered to be the major mode of HIV-1 transmission *in vivo* (reviewed in [50]). The observed large HIV-1 Env clusters may thus play an important role in viral spread and pathogenesis. Visualization of the architecture of virological synapse structures using 3D multicolor super-resolution microscopy will therefore be an important goal of future research.

## Materials and Methods

### Cell lines and plasmids

HeLa cells and A3.01 T-cells [51] were grown at 37°C and 5% CO_2_ in Dulbecco's modified Eagle's medium (DMEM; Invitrogen) and RPMI-1640 medium, respectively. Media were supplemented with 10% fetal calf serum (FCS), 100 U/ml penicillin and 100 µg/ml streptomycin. Plasmid pCHIV, expressing all HIV-1 proteins except for Nef under the control of a CMV promoter and its derivative pCHIV^mEosFP^ have been described previously [36]. pCHIVEnv(ΔCT) and pCHIV^mEosFP^. Env(ΔCT) were constructed by exchanging an AgeI/XhoI fragment of the respective parental plasmid with a corresponding fragment covering the *env* coding region from plasmid pNL4-3CTdel144-2 [14]. The HIV-1 Env expression vector pCAGGS.NL4-3-Xba, designated here as pEnv(wt), has been described previously [52]. pEnv(ΔCT) was kindly provided by Nikolas Herold. It was constructed based on pEnv(wt) by exchanging an Acc65I/XhoI restriction fragment against the corresponding fragment from an Env(ΔCT) expression vector kindly provided by Valerie Bosch [15]. HIV-1 proviral constructs carrying wt Gag or the MA L8S/S9R mutant defective in Env interaction [8], [40] in combination with either Env(wt) or or Env(ΔCT) were kindly provided by F. Mammano.

### Transfection and immunofluorescence staining

HeLa cells were seeded at a density of 1×10^4^ cells in 8-well chambered cover glasses (LabTek) and transfected with 0.2 µg of plasmid/well on the following day using the FuGene HD transfection reagent (Roche Diagnostics). Nucleofection of A3.01 cells was performed as described previously [29]. In brief: 5×10^6^ cells were electroporated with 10 µg of each plasmid in a 0.4 cm cuvette (Invitrogen) in a volume of 500 µl of serum-free medium using a Gene Pulser Xcell (BioRad). Parameters were: capacity 950 µF and 300 V. After 24 hours, cells were seeded on 8-well chambered cover glasses (LabTek) coated with fibronectin at a concentration of 5 µg/ml. After sedimentation, cells were fixed with 3% paraformaldehyde (PFA) followed by permeabilization and blocking with 2% BSA.

Two staining approaches for Env were applied: fixation followed by staining, or staining followed by fixation. For the first approach, cells were fixed at 24 h post transfection (hpt) with 3% PFA, washed and blocked for 10 min with 2% BSA in phosphate buffered saline (PBS). Harsher fixation of samples was performed using 4% PFA/0.2% glutaraldehyde for 30 min [38]. Cells were incubated with the monoclonal anti-gp120 antibody 2G12 ([35], Polymun Scientific) for 45 min. To confirm the specificity of 2G12 staining, the anti-gp120 MAb b12 ([37], Polymun Scientific) was used. The washing and blocking procedures were repeated before incubation with goat anti-human Alexa Fluor 647 secondary antibody (Invitrogen). To exclude antibody induced Env clustering and fixation artifacts, we performed staining prior to fixation and used Fab fragments of the respective antibodies (kindly provided by J. Chojnacki). All steps were performed at 16°C to block endocytic uptake of surface molecules. Cells were washed with PBS and samples were blocked with 2% BSA for 10 min, followed by incubation with 2G12 Fab for 40 min. Subsequently, cells were incubated with goat anti-human Alexa Fluor 647 Fab (Dianova) for 40 min. Stained cells were fixed with 3% PFA for 20 min and washed with PBS. Staining of the MA domain of HIV was performed using the MAb APR342 [53] (CFAR, UK) followed by goat-anti-mouse Alexa Fluor 532 antibody.

Immature eGFP.Vpr-labelled viruses [39], kindly provided by J. Chojnacki, were adhered to LabTek chamber slides coated with 5 µg/ml fibronectin, fixed with 3% PFA and stained with MAb 2G12 and goat anti-human Alexa Fluor 647 and subjected to dSTORM imaging.

### Super-resolution microscopy

Super-resolution microscopy was performed using a custom-built microscope setup as described elsewhere [54]. Briefly, a multi-line argon–krypton laser (Innova70C, Coherent, USA) and a 405 nm diode laser (Cube, Coherent, USA) were coupled into an inverted microscope (IX71, Olympus, Japan) equipped with a 63× oil immersion objective (PlanApo 63×, NA 1.45, Olympus, Japan) suitable for total internal reflection fluorescence (TIRF) imaging. The excitation and emission beams were separated using appropriate dichroic mirrors and filters (AHF, Germany). The fluorescence emission was detected by an EM-CCD camera (Ixon, Andor, Ireland).

Combined PALM and dSTORM imaging was performed sequentially, using an imaging buffer which is suitable for both photoswitching the fluorescent protein mEosFP as well as the organic fluorophore Alexa Fluor 647 or Alexa Fluor 532 [55]. Briefly, the cells were imaged in oxygen-depleted hydrocarbonate buffer (pH 8) supplemented with 100 mM mercaptoethylamine (MEA). First, Alexa Fluor 647 was reversibly photoswitched by irradiation with 488 nm (photoactivation) and 647 nm (read-out). For each channel, 8,000 to 10,000 images were recorded with an integration time of 50 ms. Then mEosFP was photoactivated by irradiation with 405 nm and imaged using an excitation wavelength of 568 nm. Alternatively, Alexa Fluor 532 was photoactivated by irradiation with 514 nm. Single-molecule localization and image reconstruction was performed using the *rapid*STORM software [56].The localization accuracy of single-molecule super-resolution microscopy was evaluated experimentally as described earlier [57] using a custom written software (Python and Scipy) [58]. For each localized fluorophore, the distance to its nearest neighbor fluorophore in an adjacent frame was calculated. As the majority of fluorophores are detected in multiple adjacent frames, the maximum of the nearest neighbor distance distribution represents the error of localization. A prerequisite for using this approach is a statistically significant number of events (n>4,000). Dual-color images were recorded by adding multi-spectral beads (Invitrogen) to the sample and post-aligning the individual images [59].

### Cluster analysis

All-distance distributions are a common tool for cluster analysis of single-molecule super-resolution data [31], [60], [61]. We used Ripley's K-function [41] (1) in its linearized (2) and normalized form (3) (Ripley's H-function):
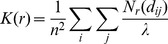
(1)

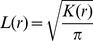
(2)

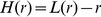
(3)where *r* is the observation radius, *n* is the total number of localizations within a region of interest (ROI), *d_ij_* is the distance between two localizations *i* and *j*, *N_r_* is the number of localizations around localization *i* within the distance *r*, and *λ* is a weighting factor correcting for the area of the ROI. We calculated Ripley's H-function for ROIs of 2×2 µm^2^. In order to account for edge effects, we used a torroidal edge correction. The results were tested against a 95% confidence envelope of uniform distributions generated by Monte Carlo methods. All calculations and simulations were performed using custom software written in Matlab (Mathworks, Natick, MA).

Image-based cluster analysis was performed on super-resolution images by identifying cohesive regions of protein populations and determining their area. For this, a ‘quantitative’ super-resolution image with a pixel size of 10 nm was generated, in which the pixel value represents the number of localizations found at this position (custom software written in Matlab (Mathworks, Natick, MA)). Cohesive regions in these quantitative images were identified and measured using an algorithm based on the analyze particles function from FIJI [62].

To analyze the protein distribution, we calculated average images by overlaying multiple images of individual clusters using an image pixel size of 5 nm. The individual images were aligned using the center of mass of the Gag protein distribution. Image averaging was performed using a custom software written in Matlab (Mathworks, Natick, USA), intensity profiles were extracted using FIJI.

## Supporting Information

Figure S1 **Experimental determination of the localization accuracy of mEosFP by a coordinate-based algorithm.** The nearest neighbor distribution of a single-molecule data set of Gag.mEosFP (n = 7,522 single-molecule localizations) was calculated. The maximum, representing localization accuracy, was found at 28 nm.
(TIF)

Figure S2 **Experimental determination of the localization accuracy of Alexa Fluor 647 by a coordinate-based algorithm.** The nearest neighbor distribution of a single-molecule data set of Alexa Fluor 647 (n = 4,196 single-molecule localizations) was calculated. The maximum, representing fluorophore localization accuracy, was found at 15 nm.
(TIF)

Figure S3 **Increased sensitivity of Gag assembly site detection.** HeLa cells were transfected with pCHIV^mEosFP^, fixed 24 hpt, stained by indirect immunofluorescence using MAb b12 and goat anti-human Alexa Fluor 647, and imaged by super-resolution TIRF microscopy as described in Materials and Methods. A representative region from the plasma membrane, showing the superposition of a PALM image for Gag.mEosFP (green) and the corresponding dSTORM image of Env stained with Alexa Fluor 647 (red), respectively. Scale bars represent 1 µm.
(TIF)

Figure S4 **Env immunostaining following harsher chemical fixation.** HeLa cells were transfected with equimolar amounts of pCHIV and pCHIV^mEosFP^. At 24 hpt cells were fixed with 4% PFA/0.2% glutaraldehyde for 30 min. Subsequently samples were stained by immunofluorescence using MAb 2G12 and goat anti-human IgG Alexa Fluor 647 and imaged by dSTORM as described in Materials and Methods. Scale bar represents 1 µm.
(TIF)

Figure S5 **Specificity of Env immunostaining.** (**A**) HeLa cells were transfected with equimolar amounts of pCHIV.Env(-) and pCHIV^mEosFP.^Env(-). Cells were fixed, immunostained using MAb 2G12 and goat anti-human IgG Alexa Fluor 647, and imaged as in Materials and Methods. (**B**) HeLa cells were transfected with an equimolar mixture of pCHIV and pCHIV^mEosFP^ and stained only with goat anti-human Alexa Fluor 647 without the primary antibody. Scale bars represent 1 µm.
(TIF)

Figure S6 **Distribution of Gag and Env at the plasma membrane of A3.01 cells analyzed by dual-color super-resolution microscopy.** (**A**) A3.01 cells were nucleofected with equimolar amounts of pCHIV and pCHIV^mEosFP^. At 24 h post nucleofection, cells were fixed, stained by indirect immunofluorescence using MAb 2G12 and goat anti-human Alexa Fluor 647 and imaged by dual-color super-resolution microscopy as described in Materials and Methods. An image of a representative cell is shown. Green, mEosFP; red, Alexa Fluor 647. Scale bar represents 2 µm. (**B**) Enlargement of three individual assembly sites from the boxed regions indicated in (A). The figure shows merged super-resolution images (left panels), the dSTORM Env Alexa Fluor 647 image (middle panels) and individual Alexa Fluor 647 localizations from all images recorded in the defined area as black dots, with a black circle representing the rims of the Gag cluster (right panels), respectively. Scale bars correspond to 100 nm. (**C**) Distribution of HIV-1 Gag and Env at the plasma membrane of A3.01 cells nucleofected with equimolar amounts of pCHIV.Env(ΔCT) and pCHIV^mEosFP^. Env(ΔCT). Cells were fixed and stained by indirect immunofluorescence using MAb 2G12 and goat anti-human Alexa Fluor 647, and subjected to dual-color super-resolution microscopy as described in Materials and Methods. Region from the plasma membrane of a representative cell, showing the superposition of a PALM image for Gag.mEosFP (green) and the corresponding dSTORM image of Env(ΔCT) stained with Alexa Fluor 647 (red), respectively. Scale bar represents 2 µm. (**D**) Enlargement of three individual assembly sites from the boxed regions indicated in (C). The figure shows merged super-resolution images (left panels), the dSTORM Env Alexa Fluor 647 image (middle panels) and individual Alexa Fluor 647 localizations from all images recorded in the defined area as black dots, with a black circle representing the rims of the Gag cluster (right panels), respectively. Scale bars correspond to 100 nm.
(TIF)

Figure S7 **Overview of Gag and Env distribution for proviral constructs expressing wt or mutated MA and wt or CT truncated Env.** Distribution of HIV-1 Gag and Env at the plasma membrane of HeLa cells transfected with the respective proviral constructs carrying both wt Gag and wt Env (**A**), wt Gag and Env(ΔCT) (**B**), Gag carrying the MA mutation and wt Env (**C**), or comprising both mutated MA and Env(ΔCT) (**D**). Expanded sections from these images highlighting individual sites are shown in main Figure 5. Scale bar represents 2 µm.
(TIF)

Figure S8 **Coordinate based all distance distribution analysis using Ripley's H-function.** The graphs show a comparison of relative Env clustering on the membrane of HeLa cells (A–D) or A3.01 cells (E–F) transfected with pCHIV/pCHIV^mEosFP^ (**A, E**), pCHIV.Env(ΔCT)/pCHIV^mEosFP^Env(ΔCT) (**B, F**), pEnv(wt) (**C**) and pEnv(ΔCT) (**D**), respectively. Two regions of interest (ROI) of 2 µm×2 µm from three cells per transfection condition were examined. The maximal H-value [nm] reflects the average cluster diameter. The amplitude of the H-function [a.u.] indicates the average degree of clustering. Six ROIs per condition were selected from the central region of the cells in order to avoid edge effects. For each ROI, Ripley's H-function was calculated. The solid red line shows the mean value for the 6 measurements and red error bars indicate the standard deviation. As a control 19 Monte Carlo simulations of a uniform distribution within a ROI were calculated in order to create a 95% confidence envelope for Ripley's H-function in case of non-cluster forming distributions (magenta and blue lines).
(TIF)

## References

[1] Sundquist WI, Krausslich HG (2012) HIV-1 Assembly, Budding, and Maturation. Cold Spring Harb Perspect Med 2: a006924. 22762019 10.1101/cshperspect.a006924 PMC3385941

[2] Checkley MA, Luttge BG, Freed EO (2011) HIV-1 envelope glycoprotein biosynthesis, trafficking, and incorporation. J Mol Biol 410: 582–608. 21762802 10.1016/j.jmb.2011.04.042 PMC3139147

[3] Chertova E, Bess JW Jr, Crise BJ, Sowder IR, Schaden TM, et al (2002) Envelope glycoprotein incorporation, not shedding of surface envelope glycoprotein (gp120/SU), Is the primary determinant of SU content of purified human immunodeficiency virus type 1 and simian immunodeficiency virus. J Virol 76: 5315–5325. 11991960 10.1128/JVI.76.11.5315-5325.2002 PMC137021

[4] Zhu P, Chertova E, Bess J Jr, Lifson JD, Arthur LO, et al (2003) Electron tomography analysis of envelope glycoprotein trimers on HIV and simian immunodeficiency virus virions. Proc Natl Acad Sci U S A 100: 15812–15817. 14668432 10.1073/pnas.2634931100 PMC307650

[5] Dorfman T, Mammano F, Haseltine WA, Gottlinger HG (1994) Role of the matrix protein in the virion association of the human immunodeficiency virus type 1 envelope glycoprotein. J Virol 68: 1689–1696. 8107229 10.1128/jvi.68.3.1689-1696.1994 PMC236628

[6] Freed EO, Martin MA (1995) Virion incorporation of envelope glycoproteins with long but not short cytoplasmic tails is blocked by specific, single amino acid substitutions in the human immunodeficiency virus type 1 matrix. J Virol 69: 1984–1989. 7853546 10.1128/jvi.69.3.1984-1989.1995 PMC188822

[7] Freed EO, Martin MA (1996) Domains of the human immunodeficiency virus type 1 matrix and gp41 cytoplasmic tail required for envelope incorporation into virions. J Virol 70: 341–351. 8523546 10.1128/jvi.70.1.341-351.1996 PMC189823

[8] Mammano F, Kondo E, Sodroski J, Bukovsky A, Gottlinger HG (1995) Rescue of human immunodeficiency virus type 1 matrix protein mutants by envelope glycoproteins with short cytoplasmic domains. J Virol 69: 3824–3830. 7745730 10.1128/jvi.69.6.3824-3830.1995 PMC189100

[9] Yu X, Yuan X, Matsuda Z, Lee TH, Essex M (1992) The matrix protein of human immunodeficiency virus type 1 is required for incorporation of viral envelope protein into mature virions. J Virol 66: 4966–4971. 1629961 10.1128/jvi.66.8.4966-4971.1992 PMC241345

[10] Cosson P (1996) Direct interaction between the envelope and matrix proteins of HIV-1. Embo J 15: 5783–5788. 8918455 PMC452325

[11] Johnson MC (2011) Mechanisms for Env glycoprotein acquisition by retroviruses. AIDS Res Hum Retroviruses 27: 239–247. 21247353 10.1089/aid.2010.0350 PMC3048835

[12] Akari H, Fukumori T, Adachi A (2000) Cell-dependent requirement of human immunodeficiency virus type 1 gp41 cytoplasmic tail for Env incorporation into virions. J Virol 74: 4891–4893. 10775630 10.1128/jvi.74.10.4891-4893.2000 PMC112014

[13] Iwatani Y, Ueno T, Nishimura A, Zhang X, Hattori T, et al (2001) Modification of virus infectivity by cytoplasmic tail of HIV-1 TM protein. Virus Res 74: 75–87. 11226576 10.1016/s0168-1702(00)00249-5

[14] Murakami T, Freed EO (2000) The long cytoplasmic tail of gp41 is required in a cell type-dependent manner for HIV-1 envelope glycoprotein incorporation into virions. Proc Natl Acad Sci U S A 97: 343–348. 10618420 10.1073/pnas.97.1.343 PMC26665

[15] Wilk T, Pfeiffer T, Bosch V (1992) Retained in vitro infectivity and cytopathogenicity of HIV-1 despite truncation of the C-terminal tail of the env gene product. Virology 189: 167–177. 1604808 10.1016/0042-6822(92)90692-i

[16] Cantin R, Methot S, Tremblay MJ (2005) Plunder and stowaways: incorporation of cellular proteins by enveloped viruses. J Virol 79: 6577–6587. 15890896 10.1128/JVI.79.11.6577-6587.2005 PMC1112128

[17] Ott DE (2008) Cellular proteins detected in HIV-1. Rev Med Virol 18: 159–175. 18265424 10.1002/rmv.570

[18] Murakami T, Freed EO (2000) Genetic evidence for an interaction between human immunodeficiency virus type 1 matrix and alpha-helix 2 of the gp41 cytoplasmic tail. J Virol 74: 3548–3554. 10729129 10.1128/jvi.74.8.3548-3554.2000 PMC111863

[19] Ono A (2010) Relationships between plasma membrane microdomains and HIV-1 assembly. Biol Cell 102: 335–350. 20356318 10.1042/BC20090165 PMC3056405

[20] Leung K, Kim JO, Ganesh L, Kabat J, Schwartz O, et al (2008) HIV-1 assembly: viral glycoproteins segregate quantally to lipid rafts that associate individually with HIV-1 capsids and virions. Cell Host Microbe 3: 285–292. 18474355 10.1016/j.chom.2008.04.004 PMC2998762

[21] Hermida-Matsumoto L, Resh MD (2000) Localization of human immunodeficiency virus type 1 Gag and Env at the plasma membrane by confocal imaging. J Virol 74: 8670–8679. 10954568 10.1128/jvi.74.18.8670-8679.2000 PMC116378

[22] Jorgenson RL, Vogt VM, Johnson MC (2009) Foreign glycoproteins can be actively recruited to virus assembly sites during pseudotyping. J Virol 83: 4060–4067. 19224995 10.1128/JVI.02425-08 PMC2668502

[23] Betzig E, Patterson GH, Sougrat R, Lindwasser OW, Olenych S, et al (2006) Imaging intracellular fluorescent proteins at nanometer resolution. Science 313: 1642–1645. 16902090 10.1126/science.1127344

[24] Heilemann M (2010) Fluorescence microscopy beyond the diffraction limit. J Biotechnol 149: 243–251. 20347891 10.1016/j.jbiotec.2010.03.012

[25] Hell SW (2007) Far-field optical nanoscopy. Science 316: 1153–1158. 17525330 10.1126/science.1137395

[26] Heilemann M, van de Linde S, Schuttpelz M, Kasper R, Seefeldt B, et al (2008) Subdiffraction-resolution fluorescence imaging with conventional fluorescent probes. Angew Chem Int Ed Engl 47: 6172–6176. 18646237 10.1002/anie.200802376

[27] Lelek M, Di Nunzio F, Henriques R, Charneau P, Arhel N, et al (2012) Superresolution imaging of HIV in infected cells with FlAsH-PALM. Proc Natl Acad Sci U S A 109: 8564–8569. 22586087 10.1073/pnas.1013267109 PMC3365178

[28] Pereira CF, Rossy J, Owen DM, Mak J, Gaus K (2012) HIV taken by STORM: super-resolution fluorescence microscopy of a viral infection. Virol J 9: 84. 22551453 10.1186/1743-422X-9-84 PMC3409066

[29] Eckhardt M, Anders M, Muranyi W, Heilemann M, Krijnse-Locker J, et al (2011) A SNAP-tagged derivative of HIV-1–a versatile tool to study virus-cell interactions. PLoS One 6: e22007. 21799764 10.1371/journal.pone.0022007 PMC3142126

[30] Lehmann M, Rocha S, Mangeat B, Blanchet F, Uji IH, et al (2011) Quantitative multicolor super-resolution microscopy reveals tetherin HIV-1 interaction. PLoS Pathog 7: e1002456. 22194693 10.1371/journal.ppat.1002456 PMC3240612

[31] Malkusch S, Muranyi W, Muller B, Krausslich HG, Heilemann M (2012) Single-molecule coordinate-based analysis of the morphology of HIV-1 assembly sites with near-molecular spatial resolution. Histochem Cell Biol 139: 173–9. 22910843 10.1007/s00418-012-1014-4

[32] Muller B, Daecke J, Fackler OT, Dittmar MT, Zentgraf H, et al (2004) Construction and characterization of a fluorescently labeled infectious human immunodeficiency virus type 1 derivative. J Virol 78: 10803–10813. 15367647 10.1128/JVI.78.19.10803-10813.2004 PMC516407

[33] Carlson LA, Briggs JA, Glass B, Riches JD, Simon MN, et al (2008) Three-dimensional analysis of budding sites and released virus suggests a revised model for HIV-1 morphogenesis. Cell Host Microbe 4: 592–599. 19064259 10.1016/j.chom.2008.10.013 PMC3454483

[34] Lampe M, Briggs JA, Endress T, Glass B, Riegelsberger S, et al (2007) Double-labelled HIV-1 particles for study of virus-cell interaction. Virology 360: 92–104. 17097708 10.1016/j.virol.2006.10.005

[35] Trkola A, Purtscher M, Muster T, Ballaun C, Buchacher A, et al (1996) Human monoclonal antibody 2G12 defines a distinctive neutralization epitope on the gp120 glycoprotein of human immunodeficiency virus type 1. J Virol 70: 1100–1108. 8551569 10.1128/jvi.70.2.1100-1108.1996 PMC189917

[36] Ivanchenko S, Godinez WJ, Lampe M, Krausslich HG, Eils R, et al (2009) Dynamics of HIV-1 assembly and release. PLoS Pathog 5: e1000652. 19893629 10.1371/journal.ppat.1000652 PMC2766258

[37] Roben P, Moore JP, Thali M, Sodroski J, Barbas CF 3rd, et al (1994) Recognition properties of a panel of human recombinant Fab fragments to the CD4 binding site of gp120 that show differing abilities to neutralize human immunodeficiency virus type 1. J Virol 68: 4821–4828. 7518527 10.1128/jvi.68.8.4821-4828.1994 PMC236421

[38] Tanaka KA, Suzuki KG, Shirai YM, Shibutani ST, Miyahara MS, et al (2010) Membrane molecules mobile even after chemical fixation. Nat Methods 7: 865–866. 20881966 10.1038/nmeth.f.314

[39] Chojnacki J, Staudt T, Glass B, Bingen P, Engelhardt J, et al (2012) Maturation-dependent HIV-1 surface protein redistribution revealed by fluorescence nanoscopy. Science 338: 524–528. 23112332 10.1126/science.1226359

[40] Monel B, Beaumont E, Vendrame D, Schwartz O, Brand D, et al (2012) HIV cell-to-cell transmission requires the production of infectious virus particles and does not proceed through env-mediated fusion pores. J Virol 86: 3924–3933. 22258237 10.1128/JVI.06478-11 PMC3302491

[41] Ripley B (1977) Modelling spatial patterns. Journal of the Royal Statistical Society 39: 172–212.

[42] Brugger B, Glass B, Haberkant P, Leibrecht I, Wieland FT, et al (2006) The HIV lipidome: a raft with an unusual composition. Proc Natl Acad Sci U S A 103: 2641–2646. 16481622 10.1073/pnas.0511136103 PMC1413831

[43] Chan R, Uchil PD, Jin J, Shui G, Ott DE, et al (2008) Retroviruses human immunodeficiency virus and murine leukemia virus are enriched in phosphoinositides. J Virol 82: 11228–11238. 18799574 10.1128/JVI.00981-08 PMC2573248

[44] Thali M (2011) Tetraspanin functions during HIV-1 and influenza virus replication. Biochem Soc Trans 39: 529–531. 21428933 10.1042/BST0390529 PMC4067976

[45] Hogue IB, Llewellyn GN, Ono A (2012) Dynamic Association between HIV-1 Gag and Membrane Domains. Mol Biol Int 2012: 979765. 22830021 10.1155/2012/979765 PMC3399408

[46] Henriksson P, Pfeiffer T, Zentgraf H, Alke A, Bosch V (1999) Incorporation of wild-type and C-terminally truncated human epidermal growth factor receptor into human immunodeficiency virus-like particles: insight into the processes governing glycoprotein incorporation into retroviral particles. J Virol 73: 9294–9302. 10516038 10.1128/jvi.73.11.9294-9302.1999 PMC112964

[47] Henriksson P, Bosch V (1998) Inhibition of cellular glycoprotein incorporation into human immunodeficiency virus-like particles by coexpression of additional cellular interaction partner. Virology 251: 16–21. 9813198 10.1006/viro.1998.9403

[48] Klein JS, Bjorkman PJ (2010) Few and far between: how HIV may be evading antibody avidity. PLoS Pathog 6: e1000908. 20523901 10.1371/journal.ppat.1000908 PMC2877745

[49] Jolly C, Kashefi K, Hollinshead M, Sattentau QJ (2004) HIV-1 cell to cell transfer across an Env-induced, actin-dependent synapse. J Exp Med 199: 283–293. 14734528 10.1084/jem.20030648 PMC2211771

[50] Sattentau QJ (2010) Cell-to-Cell Spread of Retroviruses. Viruses 2: 1306–1321. 21994681 10.3390/v2061306 PMC3185708

[51] Folks T, Benn S, Rabson A, Theodore T, Hoggan MD, et al (1985) Characterization of a continuous T-cell line susceptible to the cytopathic effects of the acquired immunodeficiency syndrome (AIDS)-associated retrovirus. Proc Natl Acad Sci U S A 82: 4539–4543. 2989831 10.1073/pnas.82.13.4539 PMC391138

[52] Bozek K, Eckhardt M, Sierra S, Anders M, Kaiser R, et al (2012) An expanded model of HIV cell entry phenotype based on multi-parameter single-cell data. Retrovirology 9: 60. 22830600 10.1186/1742-4690-9-60 PMC3464718

[53] Ferns RB, Partridge JC, Spence RP, Hunt N, Tedder RS (1989) Epitope location of 13 anti-gag HIV-1 monoclonal antibodies using oligopeptides and their cross reactivity with HIV-2. Aids 3: 829–834. 2483619 10.1097/00002030-198912000-00008

[54] Nanguneri S, Flottmann B, Horstmann H, Heilemann M, Kuner T (2012) Three-dimensional, tomographic super-resolution fluorescence imaging of serially sectioned thick samples. PLoS One 7: e38098. 22662272 10.1371/journal.pone.0038098 PMC3360663

[55] Endesfelder U, Malkusch S, Flottmann B, Mondry J, Liguzinski P, et al (2011) Chemically induced photoswitching of fluorescent probes–a general concept for super-resolution microscopy. Molecules 16: 3106–3118. 21490558 10.3390/molecules16043106 PMC6260607

[56] Wolter S, Endesfelder U, van de Linde S, Heilemann M, Sauer M (2011) Measuring localization performance of super-resolution algorithms on very active samples. Opt Express 19: 7020–7033. 21503016 10.1364/OE.19.007020

[57] Lando D, Endesfelder U, Berger H, Subramanian L, Dunne PD, et al (2012) Quantitative single-molecule microscopy reveals that CENP-A(Cnp1) deposition occurs during G2 in fission yeast. Open Biol 2: 120078. 22870388 10.1098/rsob.120078 PMC3411111

[58] Jones E, Oliphant T, Peterson P (2001) SciPy: Open source scientific tools for Python.

[59] Malkusch S, Endesfelder U, Mondry J, Gelleri M, Verveer PJ, et al (2012) Coordinate-based colocalization analysis of single-molecule localization microscopy data. Histochem Cell Biol 137: 1–10. 22086768 10.1007/s00418-011-0880-5

[60] Lillemeier BF, Mortelmaier MA, Forstner MB, Huppa JB, Groves JT, et al (2010) TCR and Lat are expressed on separate protein islands on T cell membranes and concatenate during activation. Nat Immunol 11: 90–96. 20010844 10.1038/ni.1832 PMC3273422

[61] Williamson DJ, Owen DM, Rossy J, Magenau A, Wehrmann M, et al (2011) Pre-existing clusters of the adaptor Lat do not participate in early T cell signaling events. Nat Immunol 12: 655–662. 21642986 10.1038/ni.2049

[62] Schindelin J, Arganda-Carreras I, Frise E, Kaynig V, Longair M, et al (2012) Fiji: an open-source platform for biological-image analysis. Nat Methods 9: 676–682. 22743772 10.1038/nmeth.2019 PMC3855844

[63] McDonald D, Vodicka MA, Lucero G, Svitkina TM, Borisy GG, et al (2002) Visualization of the intracellular behavior of HIV in living cells. J Cell Biol 159: 441–452. 12417576 10.1083/jcb.200203150 PMC2173076

