# Supplementary figures and images for: Super-Resolution Microscopy Reveals Specific Recruitment of HIV-1 Envelope Proteins to Viral Assembly Sites Dependent on the Envelope C-Terminal Tail
Source: PLoS Pathog. 2013 Feb 28;9(2):e1003198. doi: 10.1371/journal.ppat.1003198 (PMC3585150; doi:10.1371/journal.ppat.1003198)

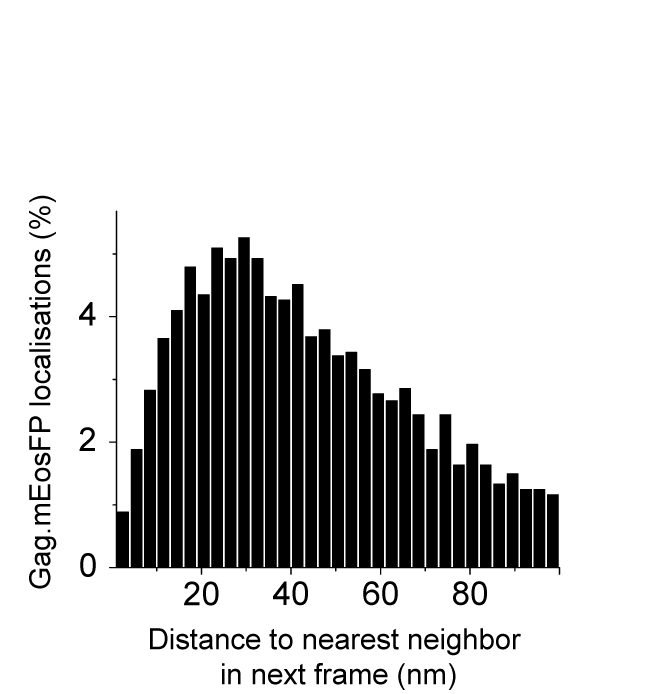

Supplement: Figure S1 — Experimental determination of the localization accuracy of mEosFP by a coordinate-based algorithm. The nearest neighbor distribution of a single-molecule data set of Gag.mEosFP (n = 7,522 single-molecule localizations) was calculated. The maximum, representing localization accuracy, was found at 28 nm. (TIF) [file ppat.1003198.s001.tif]

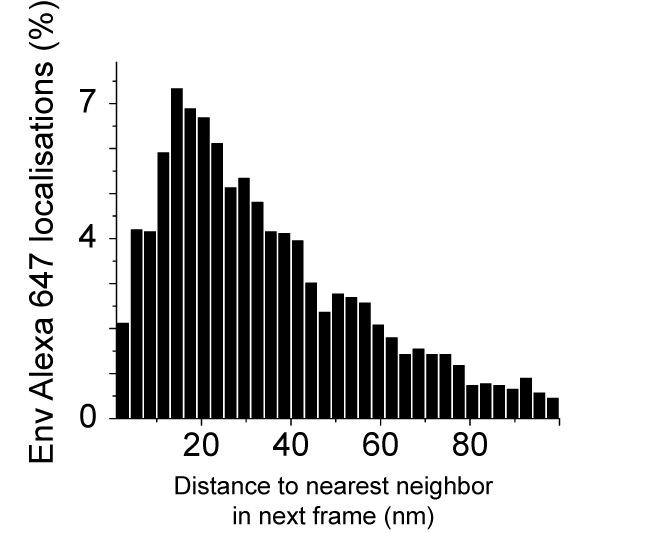

Supplement: Figure S2 — Experimental determination of the localization accuracy of Alexa Fluor 647 by a coordinate-based algorithm. The nearest neighbor distribution of a single-molecule data set of Alexa Fluor 647 (n = 4,196 single-molecule localizations) was calculated. The maximum, representing fluorophore localization accuracy, was found at 15 nm. (TIF) [file ppat.1003198.s002.tif]

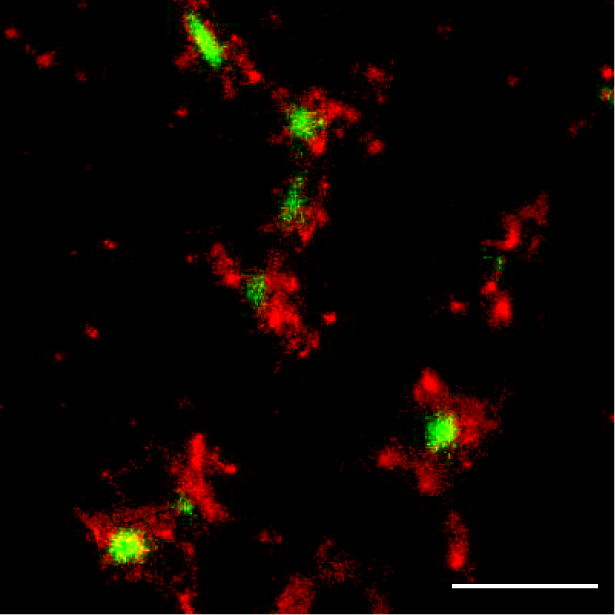

Supplement: Figure S3 — Increased sensitivity of Gag assembly site detection. HeLa cells were transfected with pCHIVmEosFP, fixed 24 hpt, stained by indirect immunofluorescence using MAb b12 and goat anti-human Alexa Fluor 647, and imaged by super-resolution TIRF microscopy as described in Materials and Methods. A representative region from the plasma membrane, showing the superposition of a PALM image for Gag.mEosFP (green) and the corresponding dSTORM image of Env stained with Alexa Fluor 647 (red), respectively. Scale bars represent 1 µm. (TIF) [file ppat.1003198.s003.tif]

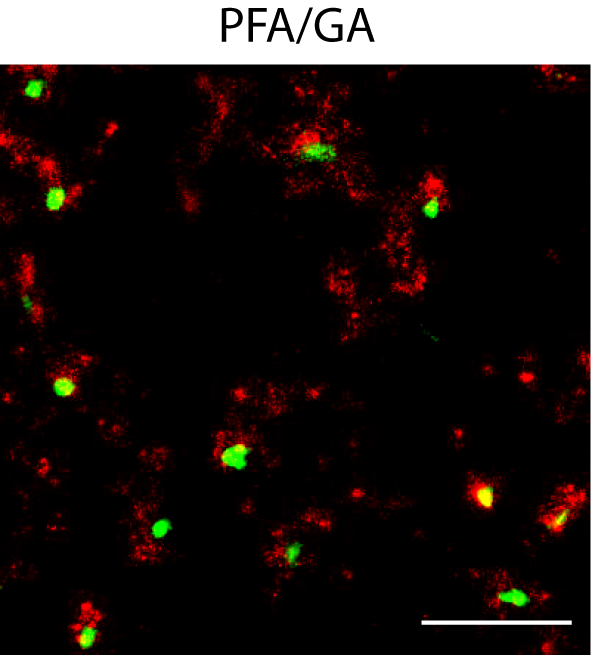

Supplement: Figure S4 — Env immunostaining following harsher chemical fixation. HeLa cells were transfected with equimolar amounts of pCHIV and pCHIVmEosFP. At 24 hpt cells were fixed with 4% PFA/0.2% glutaraldehyde for 30 min. Subsequently samples were stained by immunofluorescence using MAb 2G12 and goat anti-human IgG Alexa Fluor 647 and imaged by dSTORM as described in Materials and Methods. Scale bar represents 1 µm. (TIF) [file ppat.1003198.s004.tif]

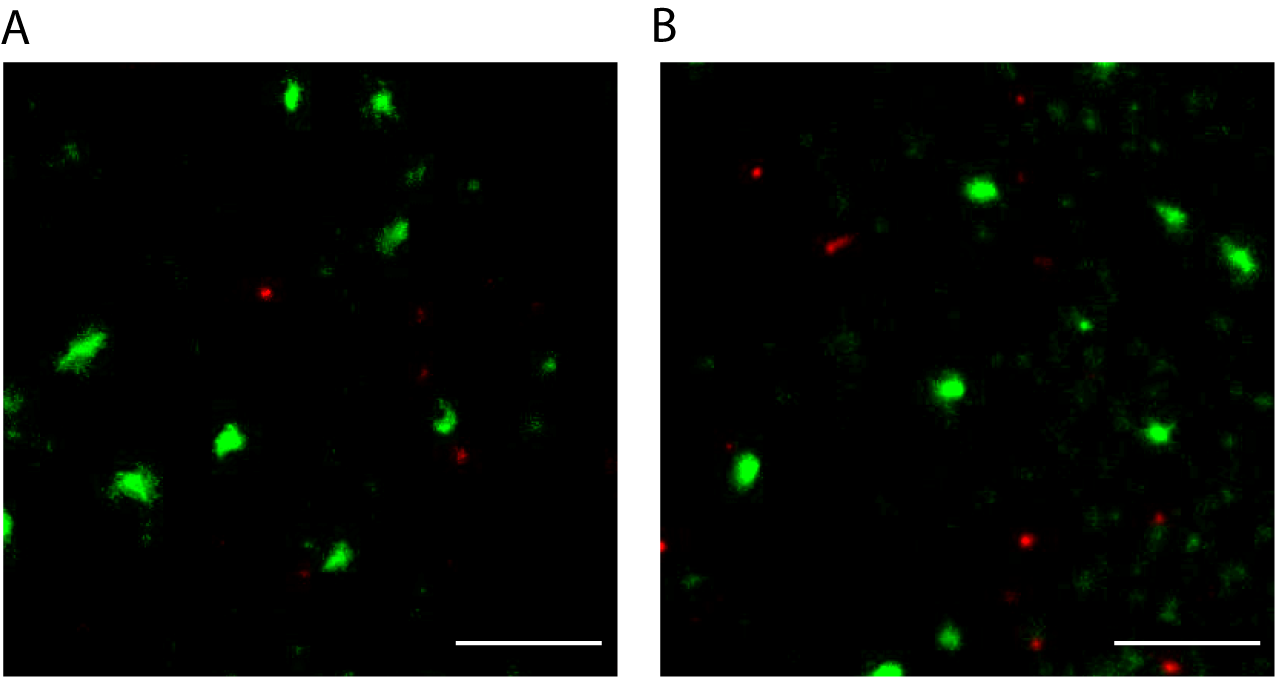

Supplement: Figure S5 — Specificity of Env immunostaining. (A) HeLa cells were transfected with equimolar amounts of pCHIV.Env(-) and pCHIVmEosFP.Env(-). Cells were fixed, immunostained using MAb 2G12 and goat anti-human IgG Alexa Fluor 647, and imaged as in Materials and Methods. (B) HeLa cells were transfected with an equimolar mixture of pCHIV and pCHIVmEosFP and stained only with goat anti-human Alexa Fluor 647 without the primary antibody. Scale bars represent 1 µm. (TIF) [file ppat.1003198.s005.tif]

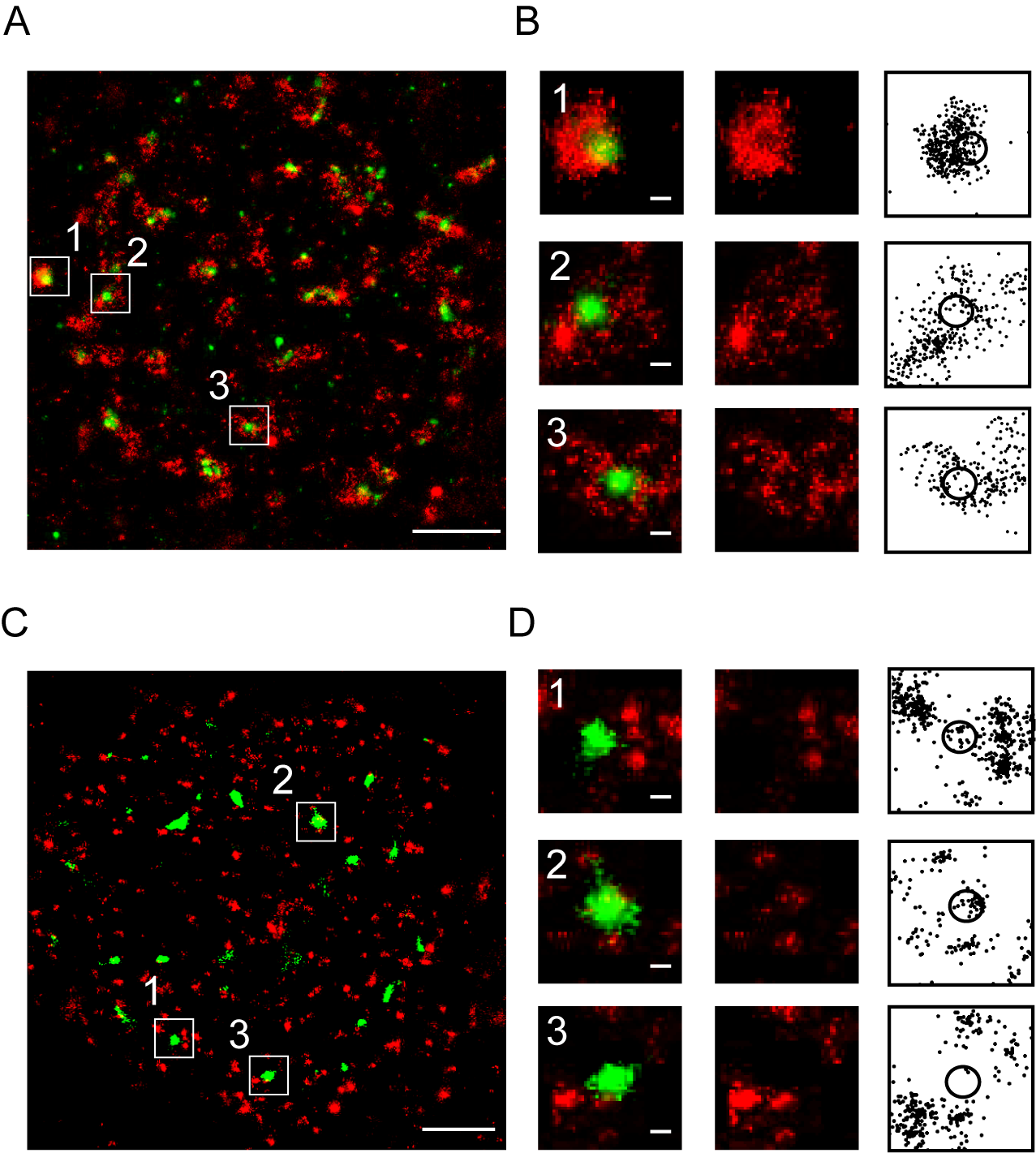

Supplement: Figure S6 — Distribution of Gag and Env at the plasma membrane of A3.01 cells analyzed by dual-color super-resolution microscopy. (A) A3.01 cells were nucleofected with equimolar amounts of pCHIV and pCHIVmEosFP. At 24 h post nucleofection, cells were fixed, stained by indirect immunofluorescence using MAb 2G12 and goat anti-human Alexa Fluor 647 and imaged by dual-color super-resolution microscopy as described in Materials and Methods. An image of a representative cell is shown. Green, mEosFP; red, Alexa Fluor 647. Scale bar represents 2 µm. (B) Enlargement of three individual assembly sites from the boxed regions indicated in (A). The figure shows merged super-resolution images (left panels), the dSTORM Env Alexa Fluor 647 image (middle panels) and individual Alexa Fluor 647 localizations from all images recorded in the defined area as black dots, with a black circle representing the rims of the Gag cluster (right panels), respectively. Scale bars correspond to 100 nm. (C) Distribution of HIV-1 Gag and Env at the plasma membrane of A3.01 cells nucleofected with equimolar amounts of pCHIV.Env(ΔCT) and pCHIVmEosFP. Env(ΔCT). Cells were fixed and stained by indirect immunofluorescence using MAb 2G12 and goat anti-human Alexa Fluor 647, and subjected to dual-color super-resolution microscopy as described in Materials and Methods. Region from the plasma membrane of a representative cell, showing the superposition of a PALM image for Gag.mEosFP (green) and the corresponding dSTORM image of Env(ΔCT) stained with Alexa Fluor 647 (red), respectively. Scale bar represents 2 µm. (D) Enlargement of three individual assembly sites from the boxed regions indicated in (C). The figure shows merged super-resolution images (left panels), the dSTORM Env Alexa Fluor 647 image (middle panels) and individual Alexa Fluor 647 localizations from all images recorded in the defined area as black dots, with a black circle representing the rims of the Gag cluster (right panels), respectively. Scale bars [file ppat.1003198.s006.tif]

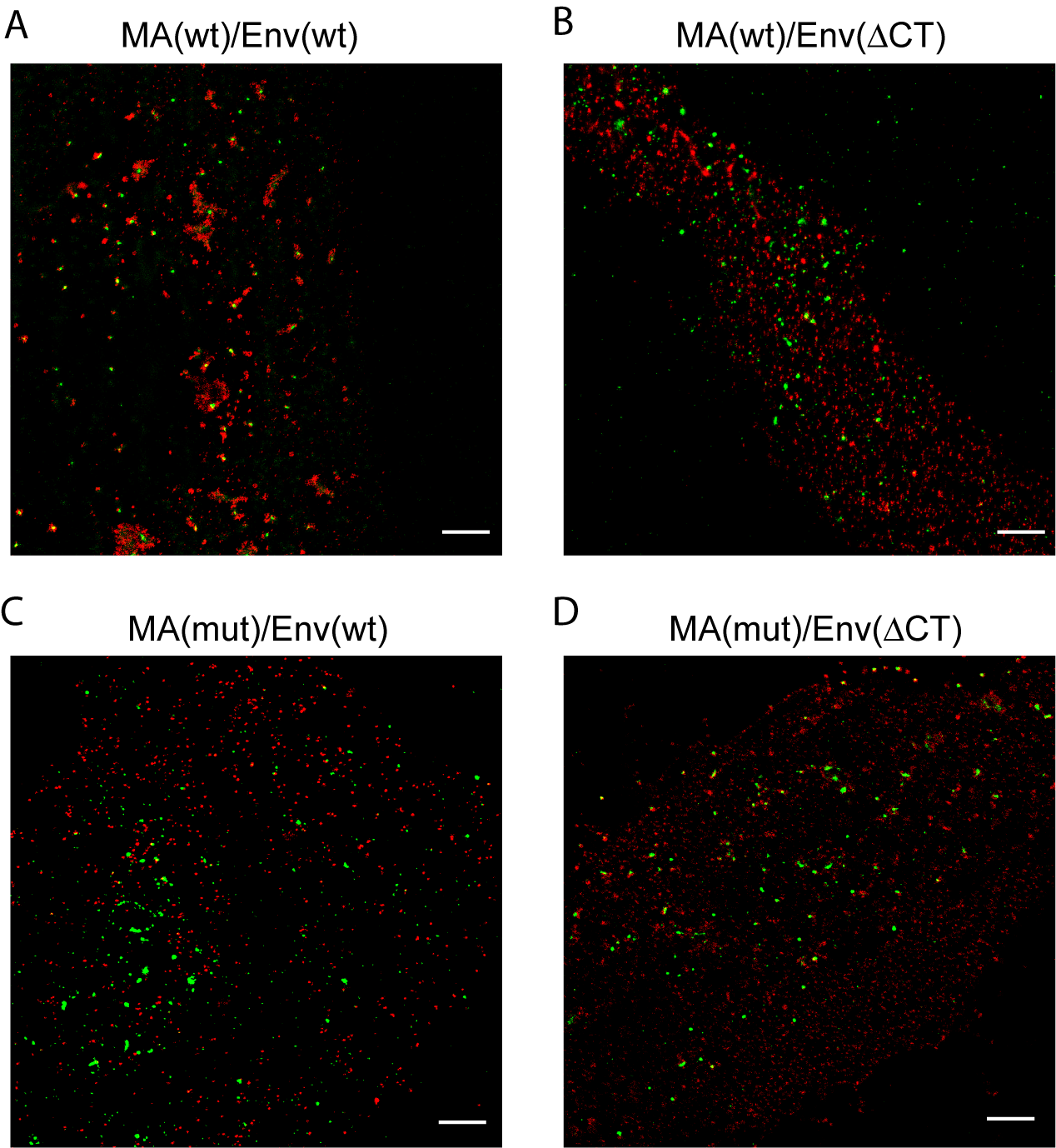

Supplement: Figure S7 — Overview of Gag and Env distribution for proviral constructs expressing wt or mutated MA and wt or CT truncated Env. Distribution of HIV-1 Gag and Env at the plasma membrane of HeLa cells transfected with the respective proviral constructs carrying both wt Gag and wt Env (A), wt Gag and Env(ΔCT) (B), Gag carrying the MA mutation and wt Env (C), or comprising both mutated MA and Env(ΔCT) (D). Expanded sections from these images highlighting individual sites are shown in main Figure 5. Scale bar represents 2 µm. (TIF) [file ppat.1003198.s007.tif]

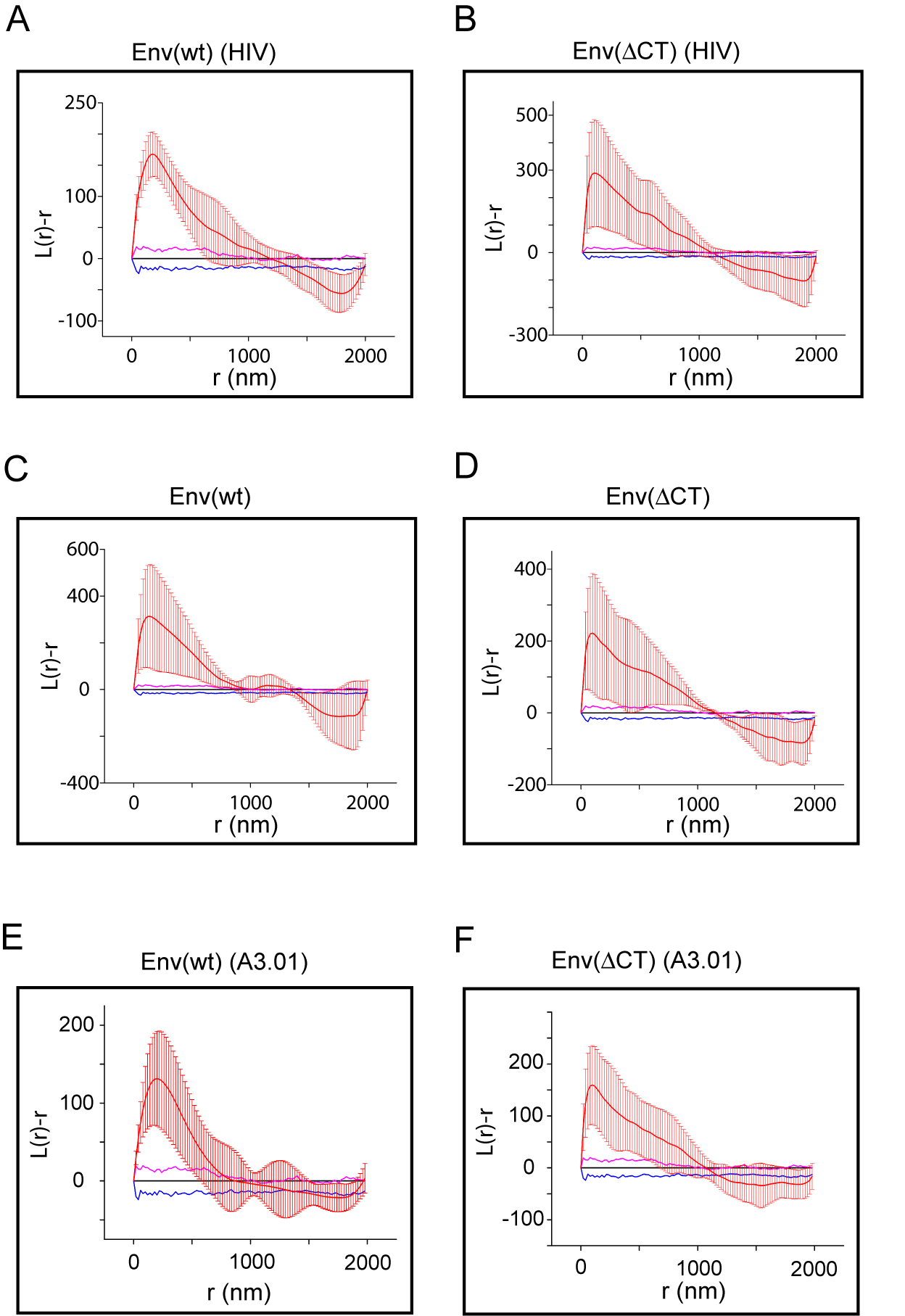

Supplement: Figure S8 — Coordinate based all distance distribution analysis using Ripley's H-function. The graphs show a comparison of relative Env clustering on the membrane of HeLa cells (A–D) or A3.01 cells (E–F) transfected with pCHIV/pCHIVmEosFP (A, E), pCHIV.Env(ΔCT)/pCHIVmEosFPEnv(ΔCT) (B, F), pEnv(wt) (C) and pEnv(ΔCT) (D), respectively. Two regions of interest (ROI) of 2 µm×2 µm from three cells per transfection condition were examined. The maximal H-value [nm] reflects the average cluster diameter. The amplitude of the H-function [a.u.] indicates the average degree of clustering. Six ROIs per condition were selected from the central region of the cells in order to avoid edge effects. For each ROI, Ripley's H-function was calculated. The solid red line shows the mean value for the 6 measurements and red error bars indicate the standard deviation. As a control 19 Monte Carlo simulations of a uniform distribution within a ROI were calculated in order to create a 95% confidence envelope for Ripley's H-function in case of non-cluster forming distributions (magenta and blue lines). (TIF) [file ppat.1003198.s008.tif]
